# Genetically Blocking the Zebrafish Pineal Clock Affects Circadian Behavior

**DOI:** 10.1371/journal.pgen.1006445

**Published:** 2016-11-21

**Authors:** Zohar Ben-Moshe Livne, Shahar Alon, Daniela Vallone, Yared Bayleyen, Adi Tovin, Inbal Shainer, Laura G. Nisembaum, Idit Aviram, Sima Smadja-Storz, Michael Fuentes, Jack Falcón, Eli Eisenberg, David C. Klein, Harold A. Burgess, Nicholas S. Foulkes, Yoav Gothilf

**Affiliations:** 1 Department of Neurobiology, George S. Wise Faculty of Life Sciences, Tel-Aviv University, Tel-Aviv, Israel; 2 Sagol School of Neuroscience, Tel-Aviv University, Tel-Aviv, Israel; 3 Institute of Toxicology and Genetics, Karlsruhe Institute of Technology, Eggenstein-Leopoldshafen, Germany; 4 Unit on Behavioral Neurogenetics, Eunice Kennedy Shriver National Institute of Child Health and Human Development, National Institutes of Health, Bethesda, Maryland, United States of America; 5 Sorbonne Universités, UPMC Univ Paris 06, CNRS, Biologie Intégrative des Organismes Marins, Observatoire Océanologique, Banyuls/Mer, France; 6 Raymond and Beverly Sackler School of Physics and Astronomy, Tel-Aviv University, Tel-Aviv, Israel; 7 Section on Neuroendocrinology and Office of the Scientific Directory, Eunice Kennedy Shriver National Institute of Child Health and Human Development, National Institutes of Health, Bethesda, Maryland, United States of America; University College London, UNITED KINGDOM

## Abstract

The master circadian clock in fish has been considered to reside in the pineal gland. This dogma is challenged, however, by the finding that most zebrafish tissues contain molecular clocks that are directly reset by light. To further examine the role of the pineal gland oscillator in the zebrafish circadian system, we generated a transgenic line in which the molecular clock is selectively blocked in the melatonin-producing cells of the pineal gland by a dominant-negative strategy. As a result, clock-controlled rhythms of melatonin production in the adult pineal gland were disrupted. Moreover, transcriptome analysis revealed that the circadian expression pattern of the majority of clock-controlled genes in the adult pineal gland is abolished. Importantly, circadian rhythms of behavior in zebrafish larvae were affected: rhythms of place preference under constant darkness were eliminated, and rhythms of locomotor activity under constant dark and constant dim light conditions were markedly attenuated. On the other hand, global peripheral molecular oscillators, as measured in whole larvae, were unaffected in this model. In conclusion, characterization of this novel transgenic model provides evidence that the molecular clock in the melatonin-producing cells of the pineal gland plays a key role, possibly as part of a multiple pacemaker system, in modulating circadian rhythms of behavior.

## Author Summary

Most physiological and behavioral processes exhibit daily rhythms which are driven by an internal timing mechanism known as the circadian clock. Circadian systems are thought to be organized in a hierarchical manner, with a central pacemaker in the brain regulating peripheral clocks throughout the body. In fish species, the pineal gland has been considered to function as a central circadian clock organ. Nevertheless, a central role for the pineal gland in the zebrafish circadian system has been questioned, because peripheral clocks in this species are independently synchronized by light. Here we developed a genetically modified zebrafish model in which the molecular clock is selectively blocked in the melatonin-producing cells of the pineal gland. As a result, clock-controlled melatonin production and gene expression in the pineal gland are interrupted. Although the independent peripheral clocks are not affected by this genetic manipulation, circadian rhythms of behavior are attenuated. These findings indicate that the zebrafish pineal gland clock contributes to the generation of daily behavioral rhythms, possibly as part of a multiple pacemaker system.

## Introduction

Numerous aspects of animal behavior and physiology vary dramatically during the course of the day-night cycle as an adaptation to recurring environmental fluctuations [1]. These variations are driven by an endogenous timing mechanism, the circadian clock, which is adjusted by external signals such as light to ensure its synchronization with the solar day [2]. At the heart of the molecular clock in vertebrates are daily oscillations in the expression and function of evolutionarily conserved clock genes and their protein products, including CLOCK and BMAL, which form heterodimers that activate the transcription of clock and clock-controlled genes (CCGs) via E-box enhancers [3].

Despite high conservation of the molecular architecture of the circadian clock, there are marked differences in organization of the circadian system among different classes of vertebrates. In mammals, neurons of the suprachiasmatic nucleus (SCN) were defined as the master circadian clock, based on the findings that: a) locomotor activity rhythms, a traditional indicator of circadian clock function, are completely lost upon SCN lesion, and b) light-induced phase shift and entrainment of behavioral rhythms to the light-dark (LD) cycle depend on photic input to the SCN [4,5]. Photic input acts on the SCN through the retinohypothalamic tract to synchronize rhythmic neuronal activity [2,4,5]. Signals from the SCN regulate circadian rhythms of multiple targets, including synthesis of the hormone melatonin in the pineal gland [6] and the synchronization and coordination of cell-autonomous molecular circadian oscillators, known as peripheral clocks, found in most tissues [7,8]. The mammalian pineal gland is regulated by the SCN via a multisynaptic pathway that leads to night-time sympathetic norepinephrine induction of melatonin synthesis [6]. Rhythmic melatonin production constitutes a major element of the circadian system in mammals, as it contributes to the regulation of various daily and annual physiological rhythms [9].

In birds, rhythmic melatonin synthesis in the pineal gland is also regulated by the SCN via sympathetic innervation [10]. However, the avian and mammalian pineal glands differ in that the avian pineal gland can also function independently to produce melatonin rhythms which are driven by a pineal gland-intrinsic clock, and in that light directly induces phase shifts of these rhythms [11–15]. Accordingly, the avian pineal gland continues to produce circadian rhythms of melatonin when kept in culture without photic cues, and light can act directly on the cultured gland to modulate these rhythms. The functional importance of the avian pineal clock is evident from the finding that pinealectomy leads to physiological and behavioral arrhythmicity. Moreover, rhythmicity can be restored by timed melatonin administration or by pineal transplantation that confers the donor's circadian phase on the recipient [16–18]. Accordingly, the circadian system of birds is considered to consist of multiple pacemakers located in the SCN, pineal gland and retina, which interact to regulate downstream physiological and behavioral rhythms [10]. Hence, the hierarchical organization of the mammalian circadian systems is not a feature shared by all vertebrates.

The fish pineal gland, as in birds, is photoreceptive and functions independently. It contains an intrinsic circadian oscillator that drives rhythms of melatonin production even when maintained in culture, disconnected from any neuronal input. Furthermore, the fish pineal gland includes cells with retinal cone photoreceptor-like characteristics, and light directly induces a phase shift of the melatonin rhythms [19–25]. The autonomy of the fish pineal gland is further emphasized by the fact that in some fish species norepinephrine does not affect melatonin synthesis [26,27]. Thus, the fish pineal gland presents all the features of a complete circadian system, comprising a photoreceptive pathway, a molecular oscillator, and an overt rhythmic output (melatonin biosynthesis). For these reasons, and since a functional counterpart to the mammalian or avian SCN has not been identified in fish, research has focused on the fish pineal gland as a master clock organ. It should be noted, however, that studies employing pinealectomy and exogenous melatonin administration have yielded species-specific effects, pointing to variations in the contribution of the fish pineal gland and melatonin to the coordination of circadian rhythms of physiology and behavior [21,24,28,29].

In zebrafish, the outcomes of pharmacological and genetic manipulations suggest that melatonin is required for the circadian regulation of the sleep/wake cycle in this species [30–33]. Nevertheless, the presence of peripheral oscillators that are photoreceptive and directly entrainable by exposure to light [34,35] has led to the view of a decentralized zebrafish circadian system, thereby questioning the central role of the pineal gland in circadian regulation. This warrants further investigation of the influence of the pineal gland-intrinsic clock on circadian rhythms of behavior and other manifestations of circadian function.

Here we address the role of the pineal gland in the zebrafish circadian system by generating and studying a novel transgenic line in which the molecular clock has been selectively blocked in the melatonin-producing cells of the pineal gland by a dominant-negative strategy. Characterization and analysis of this transgenic model establish the function of the molecular clock in the zebrafish pineal gland, probably as part of a multicomponent clock system, in regulating circadian rhythms of behavior.

## Results

### Genetically blocking the pineal gland molecular oscillator

**Fig 1:**
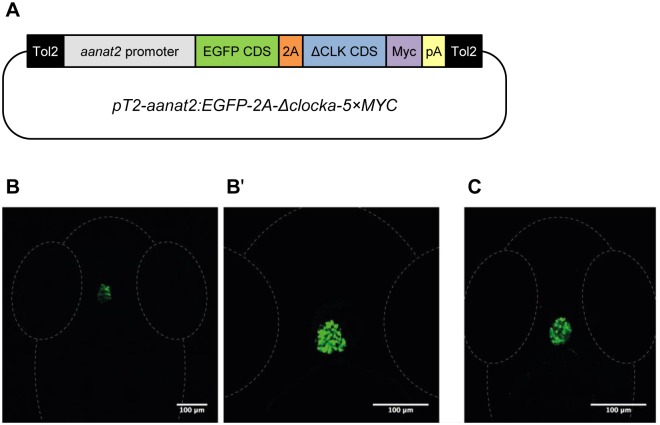
Generation of Tg(*aanat2*:EGFP-ΔCLK) fish. (A) Schematic representation of the transgenic construct used for the generation of Tg(*aanat2*:EGFP-ΔCLK) fish. The *pT2-aanat2*:*EGFP-2A-Δclocka-5×MYC* plasmid consists of two arms from the Tol2 transposable element (black), *aanat2* regulatory regions (gray), EGFP coding sequence (CDS; green), 2A peptide sequence (orange), ΔCLK CDS (blue), 5×Myc tags (purple) and SV40 poly(A) signal (yellow). (B and B') EGFP expression is restricted to the pineal gland of Tg(*aanat2*:EGFP-ΔCLK) larvae. Dorsal views of the head region of 7-day post-fertilization (dpf) larvae, anterior to the top; confocal z-stack projection (B) and a single confocal plane (B'). (C) Immunostaining with anti-Myc antibody confirms that ΔCLK is specifically expressed in the pineal gland of Tg(*aanat2*:EGFP-ΔCLK) larvae. Dorsal view of the head region of a 5-dpf larva, anterior to the top; confocal z-stack projection.

To selectively block the core molecular circadian clock of the pineal gland we generated a transgenic zebrafish line, Tg(*aanat2*:EGFP-ΔCLK) (Fig 1), in which this clock is blocked by means of a dominant-negative strategy. This line expresses a C-terminal-truncated form of the zebrafish CLOCKa protein (ΔCLK) in the melatonin-producing cells of the pineal gland, under the control of the regulatory regions of the *aanat2* gene [36], which drive specific expression in these cells. The ΔCLK mutation was originally described in mice [37] and an equivalent mutation was later exploited to transiently block the clock in zebrafish embryos [38]. The ΔCLK protein consists of the bHLH and PAS domains, enabling it to heterodimerize with BMAL and bind to the E-box enhancer sequence, but it lacks the C-terminal glutamine-rich transactivation domain, thus abolishing its capacity to activate transcription. Therefore, ΔCLK displays a dominant-negative function by competing with endogenous CLOCK proteins [38]. The dominant-negative function of ΔCLK is evident in zebrafish Pac-2 cells, in which the expression of ΔCLK abolishes rhythmic expression driven by E-box elements (S1 Fig). The Tg(*aanat2*:EGFP-ΔCLK) line also expresses enhanced green fluorescent protein (EGFP), which facilitates the identification of positive transgenics and the dissection of ΔCLK-expressing pineal glands (Fig 1B and 1B'). EGFP is separated from ΔCLK by the 2A peptide linker for production of two separate proteins [39]. Whole-mount immunostaining analysis confirms that Myc tag-labeled ΔCLK expression is restricted to the pineal gland (Fig 1C). RNA-seq analysis indicates that ΔCLK is highly expressed in the adult Tg(*aanat2*:EGFP-ΔCLK) pineal gland (30-fold higher than the endogenous *clock* genes) in a non-rhythmic manner (chart C in S2 Fig). Advantages of this dominant-negative approach over gene knockout are tissue specificity and the ability to overcome possible gene redundancy resulting from the presence of multiple *clock* paralogs in zebrafish.

### Clock-controlled pineal *aanat2* mRNA rhythm is abolished in Tg(*aanat2*:EGFP-ΔCLK) larvae

**Fig 2:**
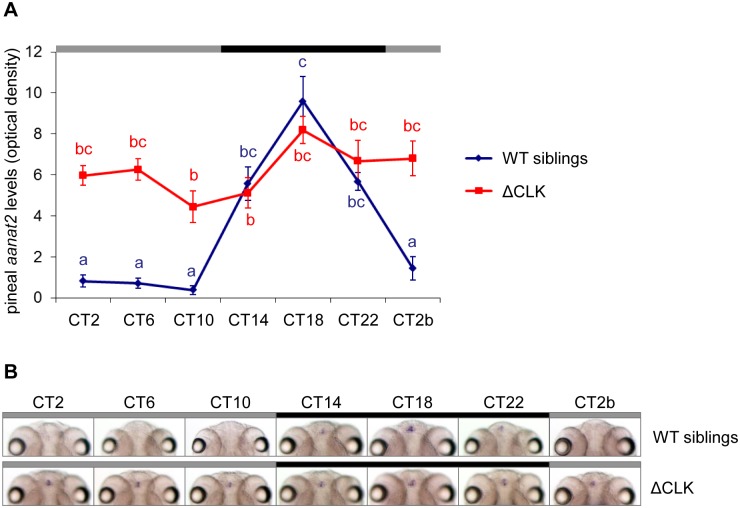
Clock-controlled *aanat2* mRNA rhythm is abolished in the pineal gland of Tg(*aanat2*:EGFP-ΔCLK) larvae. (A) Tg(*aanat2*:EGFP-ΔCLK) larvae (ΔCLK) exhibit arrhythmic pineal *aanat2* mRNA levels under DD compared with a robust rhythm in their WT siblings (*p*<0.0001, two-way ANOVA), indicating that the pineal gland molecular clock is disrupted. Each value represents the mean optical density ± SE of the pineal *aanat2* whole-mount ISH signal (*n* = 10−15 larvae). Different letters represent statistically different values (*p*<0.05, Tukey's test). (B) Representative whole-mount ISH *aanat2* signals (8 dpf, dorsal view) in the pineal glands of WT sibling larvae (upper panel) and Tg(*aanat2*:EGFP-ΔCLK) larvae (ΔCLK; bottom panel). Black and gray bars represent subjective night and day, respectively. CT, circadian time.

*Aanat2* encodes the enzyme that determines the rate of melatonin production in the pineal gland; a robust *aanat2* mRNA rhythm starting at 2 days post-fertilization (dpf) is considered as a marker for circadian clock function [40,41]. To test whether the pineal gland molecular clock has been blocked in Tg(*aanat2*:EGFP-ΔCLK) fish, we first examined the expression of *aanat2* in the larval pineal gland. Tg(*aanat2*:EGFP-ΔCLK) larvae and their wild-type (WT) siblings were entrained by seven LD cycles and then transferred to constant darkness (DD). Larvae were collected at 4-hr intervals throughout one daily cycle and subjected to whole-mount *in-situ* hybridization (ISH) for *aanat2* mRNA. A robust clock-controlled rhythm of *aanat2* expression was observed in the pineal glands of WT sibling larvae; this rhythm, however, was absent in Tg(*aanat2*:EGFP-ΔCLK) larvae (*p*<0.0001, two-way ANOVA), which exhibited intermediate to high basal levels of *aanat2* mRNA (Fig 2). Loss of the clock-controlled rhythm of *aanat2* expression demonstrates that the molecular clock in the pineal melatonin-producing cells of Tg(*aanat2*:EGFP-ΔCLK) fish was effectively disrupted.

### Clock-controlled rhythmic melatonin production is disrupted in the Tg(*aanat2*:EGFP-ΔCLK) pineal gland

**Fig 3:**
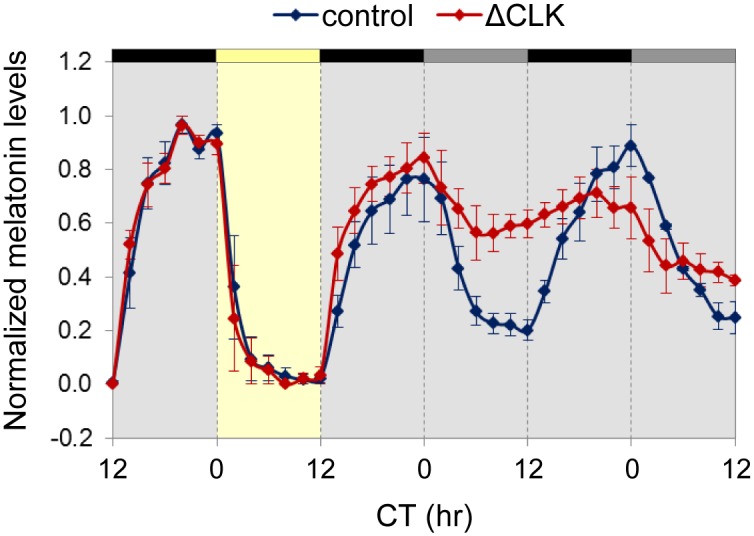
Clock-controlled rhythms of melatonin production are disturbed in ΔCLK-expressing pineal glands. Circadian rhythms of melatonin release from cultured pineal glands of Tg(*aanat2*:EGFP-ΔCLK) adult fish are disturbed under DD (*p*<0.05, Kolmogorov-Smirnov test). Yellow bar represents the light period, black bars represent dark, and gray bars represent dark during subjective day. Values indicate the normalized amount of melatonin produced by the glands. Error bars represent SE (*n* = 3). CT, circadian time.

To examine the effect of the ΔCLK mutation on rhythms of melatonin production, the pineal glands of Tg(*aanat2*:EGFP-ΔCLK) and control fish were cultured in a flow-through perfusion system. The lighting schedule was one dark-light (DL) cycle followed by two daily cycles under DD. The ΔCLK-expressing pineal glands produced a normal melatonin rhythm under the DL cycle, demonstrating that the ability to synthesize melatonin and light responsiveness were not affected by the mutation. The normal rhythmic secretion of melatonin observed in the ΔCLK-expressing pineal glands under the DL cycle reflects the effect of light on AANAT2 stability, resulting in the suppression of melatonin production. In contrast, under DD the melatonin rhythm was disrupted (*p*<0.05, Kolmogorov-Smirnov test; Fig 3), characterized by increased basal levels of melatonin release during the subjective day. These results are in accordance with the expression pattern of *aanat2*, and further validate the Tg(*aanat2*:EGFP-ΔCLK) line as a suitable model for studying the roles of the pineal clock and its primary output, melatonin, in driving circadian rhythms at the whole animal level.

### Loss of circadian expression of clock-controlled genes in the pineal glands of Tg(*aanat2*:EGFP-ΔCLK) fish

**Fig 4:**
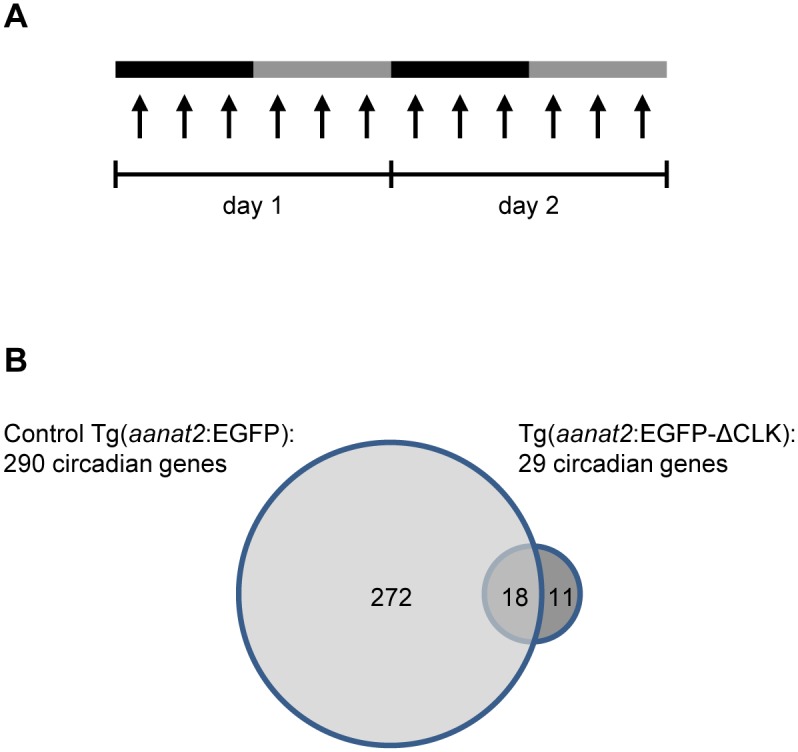
Clock-controlled rhythmic gene expression is disrupted in ΔCLK-expressing pineal glands. (A) Experimental procedure for transcriptome analysis. Adult fish were kept under DD and pineal glands were sampled at 12 time points (indicated by arrows) throughout two daily cycles. Black and gray bars correspond to subjective night and day, respectively. (B) The mRNA-seq analysis resulted in the identification of 29 circadian genes in the pineal gland of Tg(*aanat2*:EGFP-ΔCLK) fish compared with 290 circadian genes in the pineal gland of Tg(*aanat2*:EGFP) control fish.

**Fig 5:**
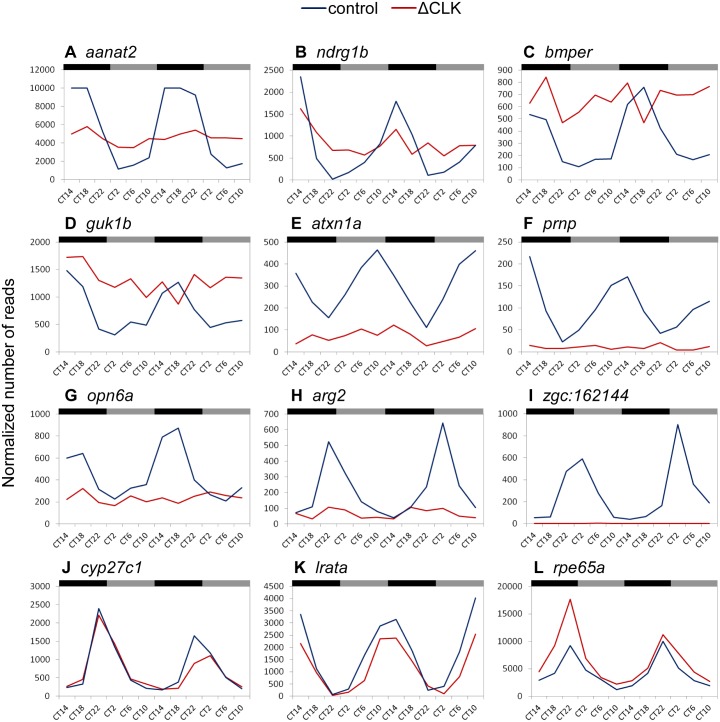
Diverse effects of ΔCLK on expression profiles of clock-controlled genes in the pineal gland. Representative examples of expression profiles of CCGs in the pineal gland of control Tg(*aanat2*:EGFP) fish (control; blue trendline) compared with the expression profiles of these genes in the pineal gland of Tg(*aanat2*:EGFP-ΔCLK) fish (ΔCLK; red trendline). Black and gray bars denote subjective night and day, respectively. CT, circadian time. While the majority of CCGs became arrhythmic (A–I), a few maintained their circadian profile in the Tg(*aanat2*:EGFP-ΔCLK) pineal gland (J–L). For some of the CCGs that became arrhythmic in the Tg(*aanat2*:EGFP-ΔCLK) pineal gland the overall basal expression levels remained relatively intermediate or high (A–D), whereas for others the expression was down-regulated (E–H) or abolished (I).

To further evaluate the effect of ΔCLK on the molecular clock in the pineal gland, we characterized circadian changes in the pineal gland transcriptome by means of mRNA-seq analysis. Pineal glands were sampled throughout two daily cycles under DD from adult Tg(*aanat2*:EGFP-ΔCLK) fish that were previously adapted to 24-hr LD cycles, replicating the experimental procedure formerly applied to Tg(*aanat2*:EGFP) fish by Tovin et al. (Fig 4A; [42]). The data obtained from mRNA-seq were subjected to Fourier analysis and compared with the data from Tg(*aanat2*:EGFP) fish that served as controls (Methods; [42]). Whereas 290 genes exhibited circadian rhythms of expression in control pineal glands, only 29 such genes were identified in Tg(*aanat2*:EGFP-ΔCLK) pineal glands (Fig 4B; S1 and S2 Tables; false-detection rate, 10%). A set of 18 genes appeared in both lists, indicating that they maintained a circadian rhythmic profile in the ΔCLK-expressing pineal glands, albeit with reduced amplitudes. These included the core clock genes *per1a*, *per1b*, *cry2a* and *cry3*, and the clock accessory loop genes *reverbb2*, *dec1* and *dec2*. In general, the circadian profiles of core clock genes and clock accessory loop genes seemed to be only partially affected by the ΔCLK mutation (S2 and S3 Figs). According to this analysis, 11 genes acquired a circadian rhythm of expression in the Tg(*aanat2*:EGFP-ΔCLK) pineal gland (S4 Fig); the regulatory mechanisms underlying the expression of these genes clearly require further examination. Importantly, the majority of CCGs lost their circadian profile in the Tg(*aanat2*:EGFP-ΔCLK) pineal gland (Fig 4B), indicating that these genes are directly or indirectly regulated by the CLOCK/BMAL heterodimer, and that the output pathways of the pineal circadian clock are substantially impaired by the ΔCLK mutation. While some CCGs that became arrhythmic display intermediate or high overall expression levels compared with their expression in the Tg(*aanat2*:EGFP) pineal gland, the expression of others is down-regulated or completely abolished, and some CCGs maintained their circadian profiles in the Tg(*aanat2*:EGFP-ΔCLK) pineal gland (Fig 5), suggesting that CCGs are regulated by various mechanisms in addition to the molecular clock.

### Global peripheral molecular clocks are not affected by the blocked pineal clock

**Fig 6:**
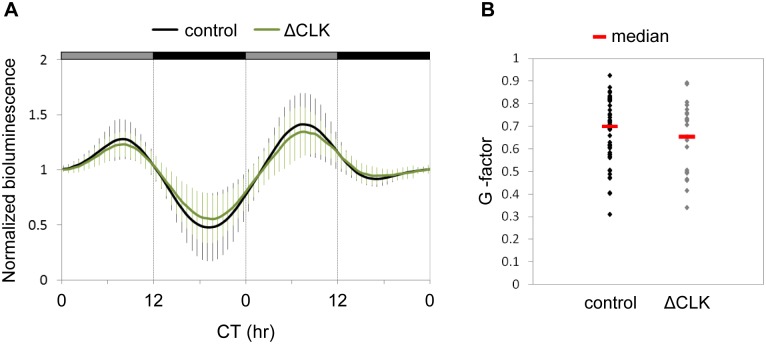
Circadian rhythms of *per1b* promoter activity in whole larvae are not affected by the pineal ΔCLK mutation. (A) Mean bioluminescence of Tg[*aanat2*:EGFP-ΔCLK;(−3.1)*per1b*::luc] larvae (ΔCLK; green trace; *n* = 23) and Tg(−3.1)*per1b*::luc larvae (control; black trace; *n* = 55), starting from 5 dpf for two daily cycles under DD. Circadian time (CT) is plotted on the x-axis. Gray and black bars represent subjective day and subjective night, respectively. Error bars represent SD. (B) Distribution of the G-factors (a representation of the extent to which the frequency of the luciferase activity pattern corresponds to a 24-hr period; see 'Fourier analysis' in S1 Text) of ΔCLK and control larvae. The median G-factor value for each group is indicated (red lines).

The role of the pineal gland oscillator in coordinating peripheral molecular clocks was analyzed using a circadian clock-reporter zebrafish line, Tg(−3.1)*per1b*::luc [43], in which a luciferase reporter is expressed under the control of the *per1b* promoter, serving as a marker for peripheral clock rhythms. Tg(*aanat2*:EGFP-ΔCLK) fish were crossed with Tg(−3.1)*per1b*::luc and the offspring, Tg[*aanat2*:EGFP-ΔCLK;(−3.1)*per1b*::luc] and control Tg(−3.1)*per1b*::luc larvae, were entrained under LD cycles and the luciferase activity in whole larvae was monitored under DD for two daily cycles. The pineal ΔCLK mutation did not significantly alter the reporter gene expression rhythms driven by the *per1b* promoter (Fig 6). This result implied that the pineal gland clock does not regulate peripheral molecular clocks, and is in accordance with the finding that peripheral clocks are not affected in melatonin-deficient fish [31]. This supports the hypothesis that peripheral clocks in zebrafish are independent of the pineal gland clock. However, this hypothesis should be taken with caution, because responses of small populations of peripheral clock-containing cells to melatonin may have been masked by the whole-larvae measurements, and because the activity of the *per1b* promoter may not be representative of the entire array of rhythmic genes in peripheral clock tissues.

### Clock-controlled rhythms of locomotor activity are affected by the blocked pineal clock

**Fig 7:**
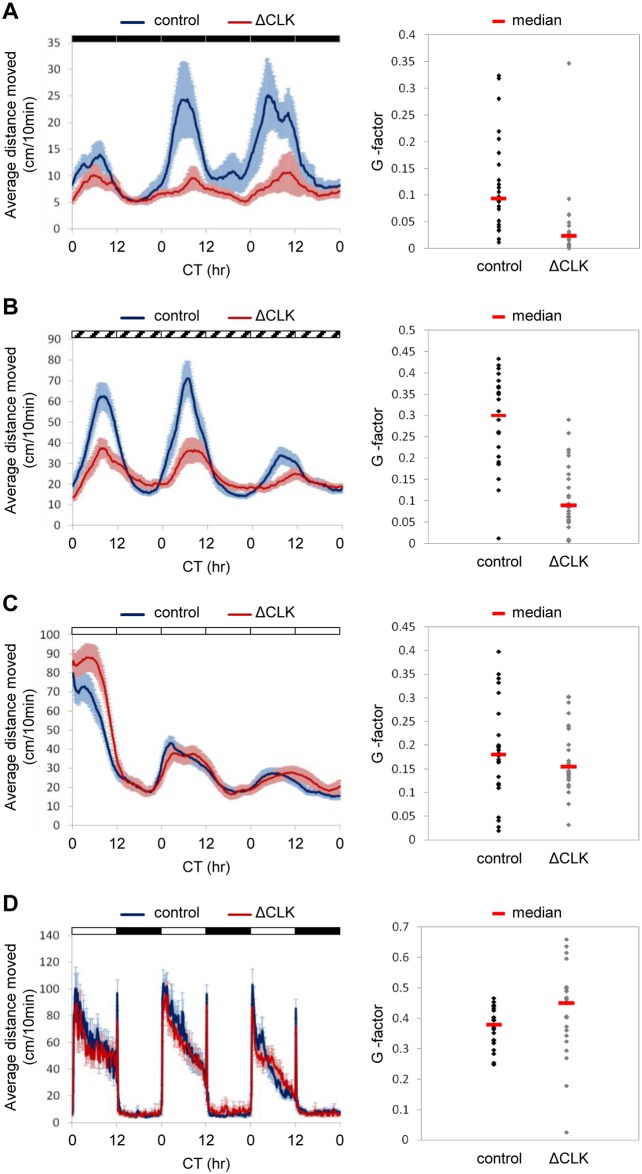
Circadian rhythms of locomotor activity under DD and DimDim, but not under LL or LD cycles, are affected by blocking the pineal clock. Analysis of locomotor activity of 6–8 dpf Tg(*aanat2*:EGFP-ΔCLK) larvae (ΔCLK) and control larvae under various lighting conditions. A–D, left chart: The average distance moved (cm/10 min) is plotted on the y-axis and circadian time (CT) is plotted on the x-axis; error bars stand for SE (*n* = 24); black, white and diagonally lined bars represent dark, light and dim light, respectively. A–D, right chart: Distribution of the G-factors (see 'Fourier analysis' in S1 Text) of Tg(*aanat2*:EGFP-ΔCLK) and control larvae; the median G-factor value for each group is indicated (red lines). (A) Circadian rhythms of locomotor activity under DD, after entrainment by 5 LD cycles, are affected by blocking the pineal clock; a significant difference in the distribution of G-factors was found between Tg(*aanat2*:EGFP-ΔCLK) and control larvae (*p*<0.0001, Kolmogorov-Smirnov test). (B) Circadian rhythms of locomotor activity under DimDim, after entrainment by 3 LD cycles and 2 light-dim light (LDim) cycles, are affected by blocking the pineal clock; a significant difference in the distribution of the G-factors was found between Tg(*aanat2*:EGFP-ΔCLK) and control larvae (*p*<0.0001, Kolmogorov-Smirnov test). (C) Circadian rhythms of locomotor activity under LL, after entrainment by 5 LD cycles, are NOT affected by blocking the pineal clock. (D) Circadian rhythms of locomotor activity under LD cycles are NOT affected by blocking the pineal clock.

To determine the role of the pineal gland clock in generating clock-regulated behavioral rhythms, we analyzed the rhythmic locomotor activity of Tg(*aanat2*:EGFP-ΔCLK) and control larvae under various photic conditions. Zebrafish larvae exhibit daily rhythms of locomotor activity under constant conditions with higher levels of activity during the subjective day, determined by prior LD cycles [44] or by a single light pulse [45]. Under LD cycles, ΔCLK expression in the pineal gland had no effect on daily rhythms of locomotor activity (Fig 7D; periods of 23.8±0.04 hr and 23.7±0.2 hr and amplitudes of 45.7±4.3 and 40.5±3 for control and ΔCLK larvae, respectively), reflecting the masking effects of light and dark. The masking effects of light and dark on clock-regulated locomotor activity of zebrafish larvae were further demonstrated under a 3.5-hr light: 3.5-hr dark schedule (S5 Fig), in which larval activity was predominantly determined by the lighting conditions. However, when LD-entrained larvae were placed under DD (Fig 7A) or constant dim light (DimDim; Fig 7B), circadian rhythms of locomotor activity were significantly affected in ΔCLK fish (*p*<0.0001, Kolmogorov-Smirnov test). Under DD, the period did not change significantly (24.9±0.6 hr and 23.7±0.8 hr for control and ΔCLK larvae, respectively), but the amplitude was significantly reduced in ΔCLK larvae (13.4±3.5 and 4.5±1.1 for control and ΔCLK larvae, respectively, *p*<0.05, *t*-test). Likewise, under DimDim both groups exhibited a similar period (23.6±0.4 hr and 24±0.5 hr for control and ΔCLK larvae, respectively), but the amplitude was significantly reduced in ΔCLK larvae (34.7±4.5 and 17±2.7 for control and ΔCLK larvae, respectively, *p*<0.01, *t*-test). Compared with DD, the use of DimDim produces higher levels of locomotor activity, higher amplitude rhythms and smaller variations in both control and Tg(*aanat2*:EGFP-ΔCLK) larvae. The residual rhythms of locomotor activity under DD or DimDim suggest that the pineal gland clock is not the sole regulator of these rhythms, and that additional clock centers, likely to be located in the central nervous system, contribute to their generation. Interestingly, under constant light (LL), the locomotor activity rhythms were not affected by the blocked pineal clock (Fig 7C; periods of 25.8±0.3 hr for both groups and amplitudes of 17.7±1.8 and 19.3±2.1 for control and ΔCLK larvae, respectively). Given that melatonin production is inhibited by light exposure and hence no melatonin is expected in either Tg(*aanat2*:EGFP-ΔCLK) or control larvae under LL, similar rhythms under these conditions hint at a possible role for melatonin in mediating the effects of the pineal gland clock on the measured behavioral outputs.

Sleep in zebrafish larvae has been defined as an inactive bout of more than 1 minute, based on the finding that larvae exhibit reduced responsiveness after 1 minute of inactivity [46]. Sleep time analysis according to this criterion resulted in effects corresponding to those of the locomotor activity analysis: The day/night sleep time differences in ΔCLK larvae were disrupted under both DD and DimDim, but not under LL or LD cycles (S6 Fig). To determine whether the reduced locomotor activity rhythms of ΔCLK larvae under DD or DimDim (Fig 7A and 7B) merely reflect increased sleep, we analyzed the activity of larvae during periods of wakefulness (waking activity; [47]). This analysis revealed that the day/night differences in waking activity are markedly reduced in ΔCLK larvae under both DD and DimDim but not under LL or LD cycles (S7 Fig). These results indicate that the effect of pineal ΔCLK expression on rhythms of locomotor activity does not simply reflect enhanced sleep.

### Circadian rhythms of position in the water column require a functional pineal clock

During sleep, animals show a preference for particular environmental locations and adopt distinctive postures. Adult zebrafish show a preference for either the top or the bottom of the tank during behavioral sleep [48]. Larval zebrafish tend to swim in the top third of the water column, rapidly descend toward the bottom of the tank upon loss of illumination [49], and remain near the bottom of the tank during behavioral sleep [33]. However, it has not been previously determined whether these changes of posture in larvae are attributable to the lighting conditions or reflect an internal circadian state. We therefore entrained larvae with LD cycles and then monitored their position in the water column for 48 hr under DD. The larvae tended to swim in the top third of the water column. However, there was a robust daily rhythm in the magnitude of this preference, with a greater proportion of larvae found in the top zone during subjective day than during subjective night (S8 Fig). Thus, as in adult zebrafish, place preference in larvae appears to be regulated by the circadian clock.

**Fig 8:**
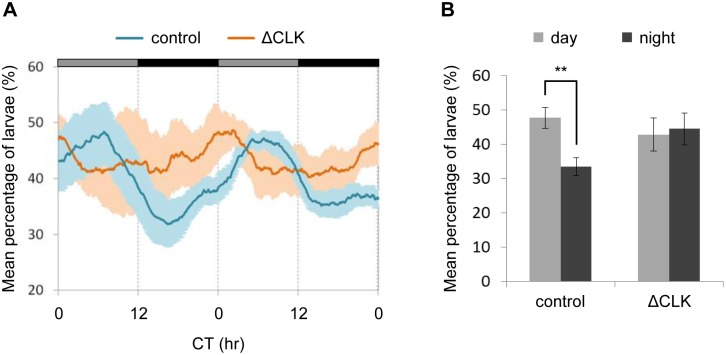
Clock-controlled rhythms of place preference are eliminated by blocking the pineal clock. Analysis of the vertical position of Tg(*aanat2*:EGFP-ΔCLK) and control larvae in the water column for two daily cycles under DD, after entrainment by 4 LD cycles. (A) The mean percentage of larvae within the top third of the water column is plotted on the y-axis and circadian time (CT) is plotted on the x-axis. Error bars represent SE [*n* = 4 groups of 15 Tg(*aanat2*:EGFP-ΔCLK) larvae, and 8 groups of 15 control larvae]. Gray and black horizontal bars represent subjective day and subjective night, respectively. A significant difference in distribution of the G-factors (see 'Fourier analysis' in S1 Text) was revealed between Tg(*aanat2*:EGFP-ΔCLK) and control larvae (*p*<0.05, Kolmogorov-Smirnov test). (B) The mean percentage of larvae in the top third of the water column at CT 5–6 (subjective day) and CT 17–18 (subjective night) is summarized for two daily cycles under DD. Significant day/night differences are observed in control larvae (**p<0.01, paired *t*-test with Bonferroni correction) but not in ΔCLK larvae.

To determine whether this rhythmic behavior is driven by the pineal gland circadian clock, Tg(*aanat2*:EGFP-ΔCLK) and control larvae were entrained by LD cycles and their position in the water column was monitored under DD for two daily cycles. As expected, control larvae displayed a clear clock-controlled rhythm of place preference, with significantly more larvae in the top third of the water column during the subjective day; however, no day/night differences in place preference were observed in Tg(*aanat2*:EGFP-ΔCLK) larvae (Fig 8). Thus, the circadian clock within the pineal gland not only modulates rhythms of locomotor activity but also drives circadian changes in place preference, suggesting that it functions as a clock center, contributing to behavioral rhythms in fish.

## Discussion

### Organization of circadian systems

The organization of circadian systems is a topic of growing interest within the field of chronobiology. It is now apparent that there are considerable differences in how multiple clocks are organized into a network, how they are synchronized, and how they impact on physiology and behavior. The organization of the circadian system in mammals is considered to be hierarchical, with the SCN being the master clock that drives essentially all circadian output rhythms [8]. This organization differs markedly from the multicomponent system seen in birds, where multiple clock centers located in the pineal gland, retina, and hypothalamus operate coordinately to regulate physiological and behavioral rhythms [10]. An important common feature of clock centers is that they can be entrained by light. The current report addresses the role of a presumed clock center in a vertebrate species possessing directly light-entrainable peripheral clocks. As discussed below, this study of a unique model, in which the clock is selectively blocked in the melatonin-producing photoreceptor cells of the pineal gland, has provided evidence that this light-sensitive clock plays an important role in the zebrafish circadian system, as it augments behavioral rhythms. Thus, the pineal gland appears to serve as a clock center for behavior; however, it is not a master oscillator like the mammalian SCN, but is rather part of a system composed of multiple clock centers, as in the case of birds.

### ΔCLK disrupts the pineal melatonin rhythm

Our findings indicate that expression of ΔCLK in the melatonin-producing cells of the pineal gland disrupts the clock-controlled rhythms of melatonin secretion (Fig 3), along with elimination of the clock-controlled mRNA rhythm of *aanat2* in the larval pineal gland (Fig 2). Notably, the overall levels of both *aanat2* expression and melatonin secretion remained intermediate to high in Tg(*aanat2*:EGFP-ΔCLK) fish, with increased subjective daytime levels compared with control fish. These results indicate that rhythms of *aanat2* expression and melatonin production are predominantly driven by the core clock mechanism. An important effect of light on melatonin production is a rapid shutdown of its synthesis through a clock-independent mechanism, in which light promotes degradation of AANAT2 protein [50–53]. Accordingly, when placed under LD cycles, the melatonin secretion pattern appears normal in Tg(*aanat2*:EGFP-ΔCLK) pineal glands (Fig 3).

### ΔCLK disrupts clock-controlled gene expression in the pineal gland

In-depth analysis of the circadian transcriptome in ΔCLK-expressing pineal glands revealed that the majority of CCGs have lost their circadian expression profile (Figs 4 and 5), indicating that the molecular clock outputs have been blocked. On the other hand, the circadian expression patterns of core clock genes and of genes considered to form clock accessory loops were only partially disturbed (S2 and S3 Figs). One possible explanation for the residual rhythmic expression of clock genes is that it derives from a functional molecular clock in neighboring pineal neuronal cells that do not produce melatonin and thus do not express ΔCLK. Another outcome of this analysis is the observation that the basal expression of the affected CCGs in the ΔCLK pineal gland ranges from being relatively high to being completely eliminated (Fig 5). This observation implies that the circadian clock regulates the rhythmic expression of CCGs, while in some cases additional factors contribute to driving their expression. Previous analysis of the zebrafish pineal circadian transcriptome showed that CCGs exhibit various phases of circadian expression [42], a finding that also points to the contribution of additional factors and mechanisms in shaping their expression patterns. It is also likely that these additional factors interact with components of the circadian clock in the regulation of target gene expression. For example, the expression of zebrafish *aanat2* was shown to be regulated by a synergistic interaction between the rhythmically expressed CLOCK/BMAL heterodimer and the photoreceptor-specific homeobox OTX5 [54].

### The pineal clock does not regulate overall peripheral molecular clock rhythms

As demonstrated by *per1b*:luc rhythms in intact larvae (Fig 6), the pineal clock and rhythmic melatonin production do not influence the global molecular rhythms of peripheral clocks. This is consistent with previous findings in melatonin-deficient larvae whose overall peripheral *per3*:luc rhythms remained unaltered [31], suggesting that in zebrafish, peripheral molecular clocks function independently of the pineal clock. However, this hypothesis should be taken with caution, for two reasons. First, the measured activity of a single promoter may not be representative of all peripheral clock and clock-controlled genes. In mice, for example, the rhythmic expression of some genes in the liver is driven by the SCN, while other genes exhibiting circadian rhythms of expression are controlled by cell-autonomous clock mechanisms [55]. Secondly, measurements of peripheral clocks carried out at the level of the whole larva may have masked the contribution of small populations of peripheral clock-containing cells that are affected by pineal-derived signals, such as melatonin. One such example in mammals is the effect of melatonin on the rhythmic expression of clock and clock-controlled genes in the pars tuberalis through the MT1 receptor [56–58]. Our knowledge of the distribution of melatonin receptors in zebrafish is limited to three melatonin receptor-encoding mRNAs, *mtnr1aa*, *mtnr1ba* and *mtnr1bb*, which appear to be widely expressed in the embryonic brain [59], two of which (*mtnr1ba* and *mtnr1bb*) are later restricted mainly to the periventricular gray zone of the optic tectum and the periventricular thalamus and hypothalamus of adult zebrafish [60]. However, the function and distribution of three other melatonin receptor-encoding genes (*mtnr1c*, *mtnr1al* and *mtnr1ab*) are still unknown. In other fish species, the expression of melatonin receptors has been shown to be widely distributed in the brain and extra-brain tissues [28,61]. Indeed, in zebrafish, exogenous melatonin was shown to gate cell proliferation during development [59], inhibit injury-induced neutrophil migration [62], modify gene expression in the liver and gonads [63,64], and directly affect ovarian oocytes [63]. These reports indicate that melatonin exerts peripheral functions in zebrafish. It remains to be determined whether certain peripheral molecular rhythms in zebrafish are driven by melatonin.

### The pineal clock contributes to circadian behavior, possibly via melatonin

Our present findings confirm that vertical positioning of zebrafish larvae in a water column is a circadian clock-regulated behavior, with a preference toward the top part of the column during the subjective daytime (S8 Fig). Under DD, circadian rhythms of positioning were absent in Tg(*aanat2*:EGFP-ΔCLK) larvae (Fig 8), indicating that this behavioral rhythm is driven by the pineal clock, thereby providing evidence for the function of the pineal gland as a behavior-regulating clock center.

Clock-regulated rhythms of locomotor activity under DD and DimDim were also significantly affected by blocking the pineal oscillator, but were not eliminated; amplitudes were significantly reduced but period length remained unaltered (Fig 7). These findings demonstrate that while the pineal clock is fundamental to augmenting activity rhythms, additional clock centers are highly likely to play a role in driving the full repertoire of circadian behaviors.

The finding that circadian control of melatonin production is absent in Tg(*aanat2*:EGFP-ΔCLK) fish is consistent with the view that in most non-mammalian vertebrates melatonin rhythms are driven by a pineal-intrinsic clock. The results of the present study support the hypothesis that melatonin is the link between the pineal clock and behavior. Locomotor activity rhythms and the corresponding sleep patterns were altered under DD and DimDim when melatonin is continuously present in Tg(*aanat2*:EGFP-ΔCLK) fish, in contrast to the rhythmicity observed in WT fish. Furthermore, this did not occur under LL, when neither of the strains produce melatonin, nor under LD cycles, when melatonin rhythms are similar in both strains (Fig 7). Similarly, decreased amplitudes of rhythmic locomotor activity were observed under DD in melatonin-deficient larvae [31], supporting the view that melatonin contributes to the regulation of these rhythms in zebrafish. The current genetic evidence for involvement of the pineal clock and its melatonin output in driving rhythms of locomotor activity and of place preference in zebrafish is supported by classical earlier studies in various fish species [28]. Nevertheless, the zebrafish pineal gland may have additional hormonal or neuronal clock outputs that regulate circadian behavior, which have yet to be investigated.

An important role for melatonin in zebrafish is in the induction of sleep. This was shown both pharmacologically by exogenous administration of melatonin [32,33], and genetically by investigation of a melatonin-deficient zebrafish line [31]. Therefore, the attenuated locomotor activity rhythms measured under DD and DimDim in ΔCLK larvae may reflect the induction of sleep by the continuously high basal levels of melatonin under these conditions. However, pineal ΔCLK expression also affected locomotor activity during periods of wakefulness (S7 Fig), suggesting that the pineal clock and possibly melatonin regulate activity rhythms independently of the somnogenic effect of melatonin.

### Future perspectives

As shown in this study, the use of a strategy in which ΔCLK is selectively expressed in a target tissue makes it possible to selectively block clock outputs while leaving the target tissue intact. This method also avoids problems encountered when interpreting gene knockout experiments, such as the existence of gene redundancy and protein moonlighting (where the targeted gene has additional, sometimes unknown, functions). This approach can potentially be used to selectively block the clock outputs in any tissue or cell type in order to study the function of specific peripheral clocks, and to further explore the organization of the circadian system and the generation of circadian behaviors in the intact animal. The nature of the additional clocks that contribute to the generation of circadian behavior still remains to be elucidated. One approach would be to genetically block the circadian clock in other specific cell subsets in the brain by the generation of additional transgenic lines that express ΔCLK under the control of various tissue-specific enhancers. These could include, for example, hypocretin neurons in the hypothalamus that have been implicated in the regulation of sleep/wake transitions in zebrafish [65], deep brain photoreceptor-expressing neurons [49], and projection neurons of the pineal gland. Thus, use of this genetic approach to study the functional significance of neuron-intrinsic circadian clocks and their importance for neuronal activity and the physiology and behavior of the intact organism can be expected to have a broad impact on the field of neurobiology.

## Methods

### Ethics statement

All procedures were approved by the Tel-Aviv University Animal Care Committee (L-10-011 and L-15-047) and conducted in accordance with the National Council for Animal Experimentation, Ministry of Health, Israel.

### Transgenic construct

The *pT2-aanat2*:*EGFP-2A-Δclocka-5×MYC* construct (Fig 1A) was generated using the backbone of the Tol2 transposable element-containing vector, pT2KXIGΔin [66]. The coding sequences of EGFP and a truncated zebrafish CLOCKa (ΔCLK; [38]) linked to 5 Myc tag sequences were cloned downstream of the *aanat2* regulatory regions [36]. A sequence encoding the 'self-cleaving' 2A peptide was cloned between the open reading frames of EGFP and ΔCLK, in order to produce two distinct proteins [39]. The sequence of the *pT2-aanat2*:*EGFP-2A-Δclocka-5×MYC* construct is provided in S9 Fig.

### Generation of transgenic fish

The transgenic line, Tg(*aanat2*:EGFP-ΔCLK), registered in the Zebrafish Model Organism Database (ZFIN) as *Tg(aanat2*:*EGFP-2A-Δclocka-5×MYC)tlv03*, was generated using the Tol2 system as described [67]. Tol2 plasmids were kindly provided by Koichi Kawakami. Capped Tol2 transposase mRNA was synthesized *in vitro* using the mMESSAGE mMACHINE SP6 Transcription Kit (Ambion) and a linearized pCS-TP plasmid as template. Approximately 1 nl of a DNA/RNA solution containing 25 ng/μl of the *pT2-aanat2*:*EGFP-2A-Δclocka-5×MYC* circular DNA and 25 ng/μl of transposase mRNA were injected into fertilized eggs at the single-cell stage. Founder (F0) fish were raised to adulthood and outcrossed to screen for integration of the transgene into the germline. Transgenic EGFP-expressing progeny (F1) were isolated using a fluorescence stereomicroscope. Several transgenic lines were obtained, all of them expressing EGFP specifically in the melatonin-producing pineal cells. F1 fish that showed strong transgene expression were further propagated. F2 progeny from one selected outcrossed F1 fish were further incrossed to generate homozygotes in the F3 generation. F3 homozygotes were further incrossed to produce homozygous transgenic progeny. WT siblings of F3 homozygotes were also maintained and further incrossed to produce control WT progeny. As in the case of transient expression of ΔCLK in embryos [38], the stable expression of ΔCLK in the pineal gland did not affect overall embryonic development.

### Whole-mount immunostaining

Tg(*aanat2*:EGFP-ΔCLK) larvae (5 dpf) were fixed, and whole-mount immunostaining was carried out as previously described [68] with mouse anti-Myc antibody 1:100 (clone 9E10; Santa Cruz Biotechnology).

### Confocal microscopy Imaging

Anesthetized or immunostained Tg(*aanat2*:EGFP-ΔCLK) larvae were placed in low melting point agarose. Images were obtained using a Leica TCS SP8 confocal laser scanning microscope equipped with Leica LAS AF image acquisition software.

### Whole-mount *in-situ* hybridization

Heterozygous Tg(*aanat2*:EGFP-ΔCLK) larvae and their WT siblings were entrained by seven 12-hr:12-hr LD cycles, transferred to DD and sampled at 4-hr intervals throughout one daily cycle. The collected embryos were fixed and subjected to whole-mount ISH followed by quantification as previously described [69,70], using the *aanat2* probe [40].

### Pineal gland perfusion system and melatonin detection

Adult fish were raised in a temperature-controlled recirculation water system under 12-hr:12-hr LD cycles and their pineal glands were collected and cultured in a flow-through system as previously described [53]. Fish were anesthetized in 1.5 mM Tricane (Sigma-Aldrich), decapitated, and pineal glands were removed by a surgical procedure carried out under a dissecting microscope (Olympus SZX12). Three pineal glands were collected from homozygous Tg(*aanat2*:EGFP-ΔCLK) fish, and three from control fish (progeny of WT siblings). The pineal glands were kept in culture medium and placed in a flow-through system. Each individual gland was placed in a glass column and continuously perfused with medium (1 ml/hr) delivered by a multi-channel peristaltic pump (Minipuls 3; Gilson). The culture medium was MEM (Sigma-Aldrich), supplemented with 2 mM L-glutamine, 0.1 mM L-tryptophan, 0.02 M sodium bicarbonate, penicillin (100,000 U/l)–streptomycin (100 mg/l) (Biological Industries) and 2.5 mg/l Fungizone (Amphotericin B; Sigma-Aldrich). The medium was continuously bubbled with a 5% CO_2_: 95% O_2_ gas mixture during the experiment. Fractions (1 ml) of medium were collected at 1-hr intervals using a multi-channel fraction collector (FC204; Gilson). The apparatus was placed inside a light- and temperature-controlled incubator and the temperature was maintained at 24°C. Pineal glands were exposed to one DL cycle followed by two DD cycles.

The concentration of melatonin in the collected medium was determined by high performance liquid chromatography (HPLC) using a 125 × 4.6 mm C18(2) reversed-phase analytic column (Phenomenex Luna) with a particle size of 5 μm and a Dionex UltiMate 3100 fluorescence detector (Thermo Scientific). A volume of 100 μl was injected from samples at 2-hr intervals. The excitation and emission wavelengths were 280 nm and 340 nm, respectively. The mobile phase consisted of 0.1 M Na_2_HPO_4_ containing 20% acetonitrile; the pH was adjusted to 6.5 with orthophosphoric acid. The mobile phase flow was 1.5 ml/min and the melatonin retention time (about 7 min) was confirmed using a commercial standard (Sigma-Aldrich).

To account for variation in the basal levels of melatonin secretion from individual pineal glands, melatonin levels secreted by each pineal gland were normalized by dividing the absolute levels by the maximal night-time levels. Levels of melatonin production by each pineal gland under DD underwent Fourier analysis and were scored with a G-factor ratio to determine circadian rhythmicity (see 'Fourier analysis' in S1 Text). Statistical differences between Tg(*aanat2*:EGFP-ΔCLK) and control fish in rhythmic melatonin production under DD were determined by the Kolmogorov-Smirnov test.

### mRNA-seq experimental procedure

Adult homozygous Tg(*aanat2*:EGFP-ΔCLK) fish were raised in a temperature-controlled recirculation water system under 12-hr:12-hr LD cycles, and transferred to DD at the end of the light period prior to sampling. Pineal glands were sampled at 4-hr intervals throughout two daily cycles under DD at 12 time points corresponding to circadian time (CT) 14, 18, 22, 2, 6, 10, 14b, 18b, 22b, 2b, 6b and 10b (Fig 4A), as previously described [42]. A pool of 16 pineal glands was collected at each time point. In addition, two control pools of 14 pineal glands were collected from Tg(*aanat2*:EGFP) fish at time points corresponding to CT2 and CT14b. Fish were anesthetized in 1.5 mM Tricane (Sigma-Aldrich) and decapitated. Fluorescent pineal glands were selectively removed under a dissecting microscope (Olympus SZX12) equipped with filters for excitation (460–490 nm) and emission (510–550 nm) of EGFP. Total RNA for mRNA analysis was isolated using RNeasy Lipid Tissue Mini Kit (Qiagen).

### mRNA-seq

mRNA-seq data acquisition and analysis were carried out as a replicate of a previously described procedure [42]. The Illumina TruSeq protocol was used to prepare libraries from RNA samples. Overall, 14 libraries [12 time points from Tg(*aanat2*:EGFP-ΔCLK) fish and two control samples from Tg(*aanat2*:EGFP) fish] were run on a single flow cell of an Illumina HiSeq2500 machine (rapid run mode) using the multiplexing strategy of the TruSeq protocol (Institute of Applied Genomics, Italy). On average, 14 million paired-end reads were obtained for each library. The reads were of 2×50 base pairs. The sequencing data were deposited in the Sequence Read Archive, under accession SRP016132. TopHat [71] was used for aligning the reads against the zebrafish genome, keeping only uniquely aligned reads with up to two mismatches per read. On average, 68% of the reads had unique alignment with the zebrafish genome. Reads aligned with the protein coding regions of known NCBI reference sequence (RefSeq) genes were used. A custom script written in Perl was used to parse the output of TopHat, which is given in Sequence Alignment/Map (SAM) format (http://samtools.sourceforge.net/), and to convert it into a raw number of reads aligned to each position in each RefSeq gene. The RefSeq genes data was obtained from the Table Browser of the UCSC genome browser (genome.ucsc.edu/) using the zebrafish July 2010 (Zv9/danRer7) assembly. To avoid PCR duplicates, only paired-end reads with unique start positions in the genome in both pairs were used [72].

### Normalization of the sequencing libraries and transcript filtering

The 26 mRNA-seq profiles (i.e., the number of reads aligned against each RefSeq gene) corresponding to the 12 time points using pineal glands of Tg(*aanat2*:EGFP-ΔCLK) fish, the same 12 time points using pineal glands of control Tg(*aanat2*:EGFP) fish (data from Tovin et al. [42]), and the two control pineal gland samples from Tg(*aanat2*:EGFP) fish (CT2 and CT14b) were normalized together. The logarithmically transformed dataset was normalized using quantile normalization [73]. Transcripts with low maximum expression values (i.e., their highest level over all time points is in the lower quartile of all transcripts) were not included in the Fourier analysis.

### Fourier analysis

Fourier analysis was conducted as previously described ([42]; S1 Text).

### Comparison of pineal gland samples from CT2 and CT14b between the different experiments

See S1 Text.

### Real-time bioluminescence assay in larvae

Tg(*aanat2*:EGFP-ΔCLK) fish were crossed with Tg(−3.1)*per1b*::luc fish [43]. Embryos were raised under 12-hr:12-hr LD cycles at 25°C. At 3 dpf, single larvae were transferred into individual wells of a 96-multiwell plate (Nunc) in E3 media (without methylene blue) supplemented with 0.5 mM beetle luciferin potassium salt solution (Promega), and the plate was sealed using an adhesive TopSeal sheet (Packard). Plates were then subjected to two LD cycles, followed by two daily cycles under DD, under which bioluminescence from whole larvae was assayed using a TopCount NXT Scintillation Counter (2-detector model; Packard). Bioluminescence data were analyzed using the Import and Analysis Macro (I&A, Plautz and Kay, Scripps) for Microsoft Excel or CHRONO software [74]. Short-term and long-term trends were removed from the raw data by an adjacent-averaging method with 3-hr and 2-day running means, respectively. Normalized values were obtained by dividing the bioluminescence values by their average value for each larva. Traces represent the mean value ± SD of 23 Tg[*aanat2*:EGFP-ΔCLK;(−3.1)*per1b*::luc] larvae and 55 control Tg(−3.1)*per1b*::luc larvae; each group was comprised of larvae from two separate crosses. To determine the circadian rhythmicity of *per1b* promotor activity, bioluminescence data from each larva underwent Fourier analysis and were scored with a G-factor ratio (see 'Fourier analysis' in S1 Text). Kolmogorov-Smirnov test was applied to compare the distribution of G-factors between Tg[*aanat2*:EGFP-ΔCLK;(−3.1)*per1b*::luc] and control Tg(−3.1)*per1b*::luc larvae.

### Transient transfection of Pac-2 cells and real-time bioluminescence assay

See S1 Text.

### Larval locomotor activity assays

Homozygous Tg(*aanat2*:EGFP-ΔCLK) embryos and control embryos (progeny of WT siblings) were raised in a light- and temperature-controlled incubator under 12-hr:12-hr LD cycles at 28°C. On the 4^th^ day of development, larvae were placed in 48-well plates in the observation chamber of the DanioVision tracking system (Noldus Information Technology) for acclimation under controlled temperature (28°C) and lighting conditions (LED; intensity of 'light' and 'dim light' were 1.8 W/m^2^ and 0.013 W/m^2^, respectively) according to the desired protocol. Starting from the 6^th^ day of development, movement was tracked and analyzed by the Ethovision 11.0 software (Noldus Information Technology).

For the analysis of locomotor activity, the raw data were converted into the total distance moved (cm) by each larva per 10 min time-bins. The data are presented as a moving average (20 sliding points) of 24 larvae in each group, excluding experiments with light-dark or dark-light transitions that trigger a temporary rise in activity (Fig 7D and S4 Fig). To determine alterations in circadian rhythms of locomotor activity, individual tracks underwent Fourier analysis and were scored with a G-factor ratio (see 'Fourier analysis' in S1 Text). Differences in G-factor distributions between Tg(*aanat2*:EGFP-ΔCLK) and control groups were determined by the Kolmogorov-Smirnov test. The periods of locomotor activity rhythms were computed by the chi-square periodogram [75] with ActogramJ software ([76]; http://actogramj.neurofly.de/), and statistical differences between Tg(*aanat2*:EGFP-ΔCLK) and control larvae were determined by *t*-test. Amplitude values were calculated as the difference between the peak of activity at day 7 and the preceding trough, divided by 2, and statistical differences between Tg(*aanat2*:EGFP-ΔCLK) and control larvae were determined by *t*-test. For analyzing the experiment in which activity was monitored under 3.5-hr light: 3.5-hr dark cycles (masking protocol, S5 Fig), the percentage of activity during the bouts of light and dark was calculated, and the difference between Tg(*aanat2*:EGFP-ΔCLK) and control larvae was determined by *t-*test.

For the analysis of sleep and waking activity, the raw data were converted to the number of seconds spent moving per 1-min time bin for each larva, with stop velocity threshold of 0.59 cm/s and start velocity threshold of 0.6 cm/s [65]. Sleep time was calculated as the number of minutes without movement per 1-hr. Waking activity was computed as the average number of seconds of activity/waking minutes per 1-hr. Data are presented as the average sleep time or average waking activity of 24 larvae in each group, and also as the average sleep time or average waking activity during subjective day and subjective night periods. Repeated-measures ANOVA was applied to compare the sleep time and log-transformed waking activity values of Tg(*aanat2*:EGFP-ΔCLK) and control groups during subjective day and subjective night periods.

### Larval vertical positioning assay

For place preference, larvae were tested in groups of 15 in chambers of 15 mm × 22 mm × 50 mm (width, length, height). Larvae were initially entrained under LD cycles for 4 days, and were then placed in the chamber under DD. Recordings were initiated after a 24-hr acclimation period, using a μEye IDS-1545LE-M CMOS camera (1stVision) to capture a snapshot of each group every 15 sec. Backlit illumination was provided by an 880-nm infrared LED array (Advanced Illumination) which was activated for 50 ms, synchronized with image acquisition. Timing was controlled by DAQtimer event control software and image analysis to detect the position of larvae performed using FLOTE [77]. For analysis, we then calculated the proportion of larvae in each of three identically sized zones of the chamber: top, middle and bottom. For behavioral testing we used homozygous Tg(*aanat2*:EGFP-ΔCLK) larvae, and progeny of WT siblings as controls.

The raw data were converted into the average percentage of larvae in the top third of the water column per 5-min time-bins. The data are presented as a moving average (40 sliding points) of four groups of Tg(*aanat2*:EGFP-ΔCLK) larvae and eight groups of control larvae. To determine alterations in circadian rhythms of environmental positioning, the data underwent Fourier analysis and were scored with a G-factor ratio (see 'Fourier analysis' in S1 Text). Differences in the G-factor distributions between Tg(*aanat2*:EGFP-ΔCLK) and control groups were determined by the Kolmogorov-Smirnov test. Differences in the average percentage of larvae in the top third of the water column between CT 5–6 (subjective day) and CT 17–18 (subjective night), under two daily cycles, were determined by paired *t*-test.

## Supporting Information

S1 Fig Expression of ΔCLK abolishes the clock-controlled rhythm driven by E-box elements in Pac-2 cells. Bioluminescence assay of zebrafish photosensitive Pac-2 cells transiently cotransfected with ΔCLK and *E-box*_*per1b*_*-Luc*. Normalized luciferase activity is plotted on the y-axis and time (days) is plotted on the x-axis. White and black bars represent the light and dark periods, respectively. Error bars represent SD.
(TIF)

S2 Fig Expression profiles of core clock genes in the pineal gland as detected by mRNA-seq analysis. (A–M) Expression profiles of core clock genes that were identified as circadian in the pineal gland of control Tg(*aanat2*:EGFP) fish (control; blue trendline) compared with their expression profiles in the pineal gland of Tg(*aanat2*:EGFP-ΔCLK) fish (ΔCLK; red trendline). Black and gray bars denote subjective night and day, respectively. The highly elevated expression of *clocka* in the Tg(*aanat2*:EGFP-ΔCLK) pineal gland (C, right vertical axis) reflects overexpression of the transgenic truncated form (ΔCLK). CT, circadian time. The core clock genes *per1a*, *per1b*, *cry2a* and *cry3* (F, G, J, L) were identified as circadian in the pineal gland of Tg(*aanat2*:EGFP-ΔCLK) fish while all other core clock genes lost their circadian rhythmicity, according to Fourier analysis with 90% true-positive rate.
(TIF)

S3 Fig Expression profiles of clock accessory loop genes in the pineal gland as detected by mRNA-seq analysis. (A–L) Expression profiles of clock-controlled genes that are considered to form accessory loops of the molecular circadian oscillator, and which were identified as circadian in the pineal gland of control Tg(*aanat2*:EGFP) fish (control; blue trendline), compared with their expression profiles in the pineal gland of Tg(*aanat2*:EGFP-ΔCLK) fish (ΔCLK; red trendline). Black and gray bars denote subjective night and day, respectively. CT, circadian time. The clock accessory loop genes *reverbb2*, *dec1* and *dec2* (D, K, L) were identified as circadian in the pineal gland of Tg(*aanat2*:EGFP-ΔCLK) fish, whereas, according to Fourier analysis with a 90% true-positive rate, other clock accessory loop genes lost their circadian rhythmicity.
(TIF)

S4 Fig Eleven genes acquired a circadian expression profile in the ΔCLK-expressing pineal gland. (A–C) Three representative examples of genes that were identified as circadian in the pineal gland of Tg(*aanat2*:EGFP-ΔCLK) fish (ΔCLK; red trendline) but not in the pineal gland of control Tg(*aanat2*:EGFP) fish (control; blue trendline). Black and gray bars denote subjective night and day, respectively. CT, circadian time.
(TIF)

S5 Fig Locomotor activity in zebrafish larvae is masked by light and dark. Analysis of locomotor activity of 6–8 dpf Tg(*aanat2*:EGFP-ΔCLK) larvae (ΔCLK) and control larvae under ten 3.5-hr light: 3.5-hr dark cycles after entrainment by five 12-hr:12-hr LD cycles. (A) The average distance moved (cm/10 min) is plotted on the y-axis and circadian time (CT) on the x-axis. Error bars stand for SE (*n* = 24). White and black bars represent light and dark, respectively. (B) Average percentage (± SE, *n* = 24) of activity throughout the bouts of light and dark for each group. No significant difference in the average percentage of activity was found between Tg(*aanat2*:EGFP-ΔCLK) and control larvae under light and dark conditions.
(TIF)

S6 Fig Circadian rhythms of sleep under DD and DimDim, but not under LL and LD cycles, are affected by blocking the pineal clock. Sleep analysis of 6–8 dpf Tg(*aanat2*:EGFP-ΔCLK) larvae (ΔCLK) and control larvae under various lighting conditions. A–D, left chart: The average sleep time (min/hr) is plotted on the y-axis and circadian time (CT) on the x-axis. Error bars stand for SE (*n* = 24); black, white and diagonally lined bars represent dark, light and dim light, respectively. A–D, right chart: The average sleep time (± SE, *n* = 24) for total subjective daytime and total subjective nighttime for each group. (A) Circadian rhythms of sleep under DD, after entrainment by 5 LD cycles, are affected by blocking the pineal clock; significant differences in day/night sleep time alterations were found between Tg(*aanat2*:EGFP-ΔCLK) and control larvae (*p*<0.05, repeated-measures ANOVA). (B) Circadian rhythms of sleep under DimDim, after entrainment by 3 LD and 2 Ldim cycles, are affected by blocking the pineal clock; significant differences in day/night sleep time alterations were found between Tg(*aanat2*:EGFP-ΔCLK) and control larvae (*p*<0.0001, repeated-measures ANOVA). (C) Circadian rhythms of sleep under LL, after entrainment by 5 LD cycles, are NOT affected by blocking the pineal clock. (D) Circadian rhythms of sleep under LD cycles are NOT affected by blocking the pineal clock.
(TIF)

S7 Fig Circadian rhythms of waking activity under DD and constant DimDim, but not under LL and LD cycles, are affected by blocking the pineal clock. Waking activity analysis of 6–8 dpf Tg(*aanat2*:EGFP-ΔCLK) larvae (ΔCLK) and control larvae under various lighting conditions. A–D, left chart: The average waking activity (sec/min) is plotted on the y-axis and circadian time (CT) on the x-axis; error bars stand for SE (*n* = 24); black, white and diagonally lined bars represent dark, light and dim light, respectively. A–D, right chart: The average waking activity (±SE, *n* = 24) for total subjective daytime and total subjective nighttime for each group. (A) Circadian rhythms of waking activity under DD, after entrainment by 5 LD cycles, are affected by blocking the pineal clock; significant difference in the day/night waking activity alterations was found between Tg(*aanat2*:EGFP-ΔCLK) and control larvae (*p*<0.001, repeated-measures ANOVA). (B) Circadian rhythms of waking activity under DimDim, after entrainment by 3 LD and 2 Ldim cycles, are affected by blocking the pineal clock; significant differences in the day/night waking activity alterations were found between Tg(*aanat2*:EGFP-ΔCLK) and control larvae (*p*<0.001, repeated-measures ANOVA). (C) Circadian rhythms of waking activity under LL, after entrainment by 5 LD cycles, are NOT affected by blocking the pineal clock. (D) Circadian rhythms of waking activity under LD cycles are NOT affected by blocking the pineal clock.
(TIF)

S8 Fig Circadian regulation of vertical place preference in zebrafish larvae. After entrainment to 4 LD cycles, larvae maintained in DD show a daily oscillation in their vertical position in the water column. (A) Mean percentage of larvae in the top, middle and bottom thirds of the water column is plotted on the y-axis and circadian time (CT) on the x-axis. Error bars stand for SE (*n* = 12). Gray and black horizontal bars represent subjective day and subjective night, respectively. (B) Distribution of the G-factors (see 'Fourier analysis' in S1 Text) of the percentage of larvae in the top, middle and bottom thirds of the water column. The median G-factor values are indicated (black lines). Since the oscillation is most prominent in the top third of the water column, it was selected as the measure for this assay.
(TIF)

S9 Fig Detailed sequence of the *pT2-aanat2*:*EGFP-2A-Δclocka-5×MYC* transgenic construct. Color coding as indicated.
(PDF)

S10 Fig True-positive rate as a function of the number of circadian transcripts detected. (A) Control Tg(*aanat2*:EGFP) dataset. (B) Tg(*aanat2*:EGFP-ΔCLK) dataset. Red circles denote the list length for 90% true-positive rate.
(TIF)

S1 Table Circadian genes in the pineal gland of Tg(*aanat2*:EGFP-ΔCLK) fish. (XLSX)

S2 Table Circadian genes in the pineal gland of control Tg(*aanat2*:EGFP) fish. (XLSX)

S1 Text Supplementary Methods. (PDF)
