# Supplementary figures and images for: Genetically Blocking the Zebrafish Pineal Clock Affects Circadian Behavior
Source: PLoS Genet. 2016 Nov 21;12(11):e1006445. doi: 10.1371/journal.pgen.1006445 (PMC5147766; doi:10.1371/journal.pgen.1006445)

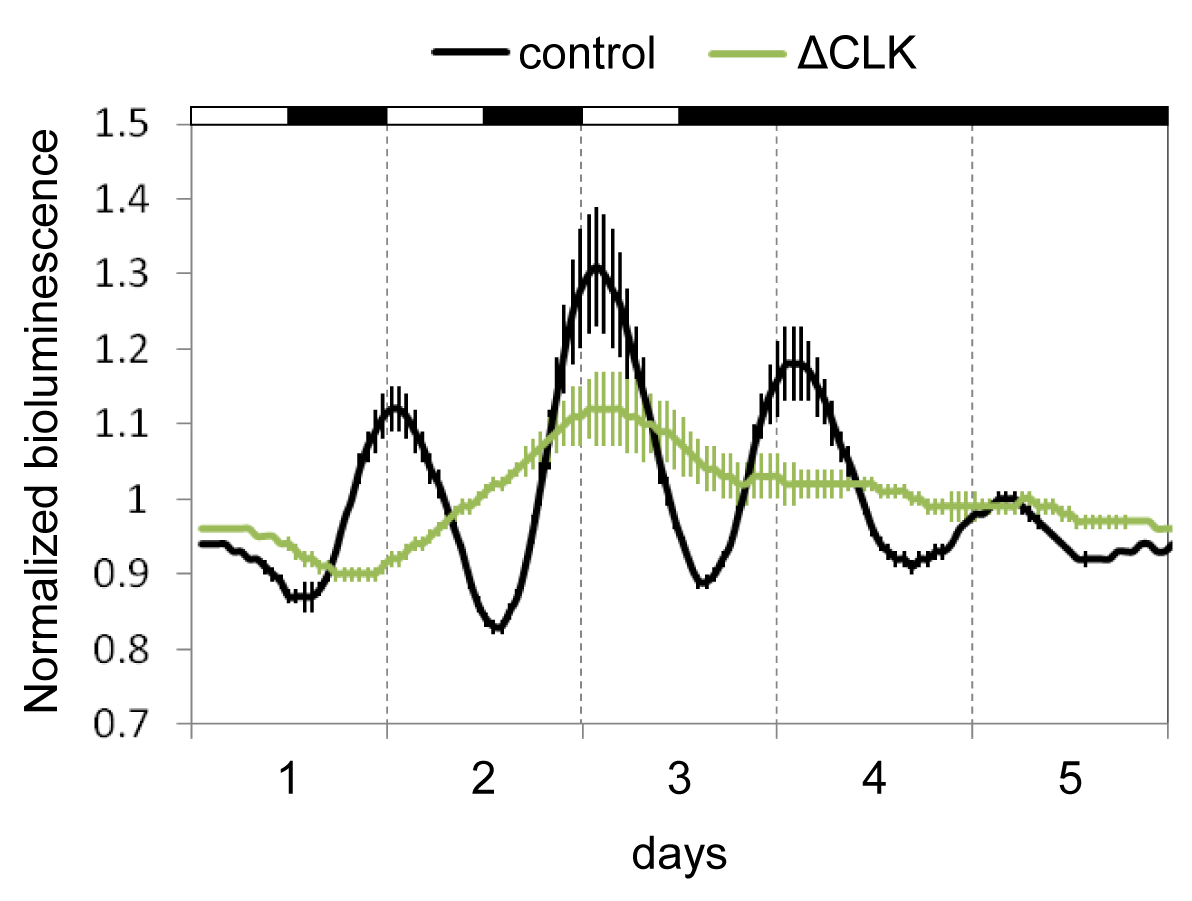

Supplement: S1 Fig — Bioluminescence assay of zebrafish photosensitive Pac-2 cells transiently cotransfected with ΔCLK and E-boxper1b-Luc. Normalized luciferase activity is plotted on the y-axis and time (days) is plotted on the x-axis. White and black bars represent the light and dark periods, respectively. Error bars represent SD. (TIF) [file pgen.1006445.s001.tif]

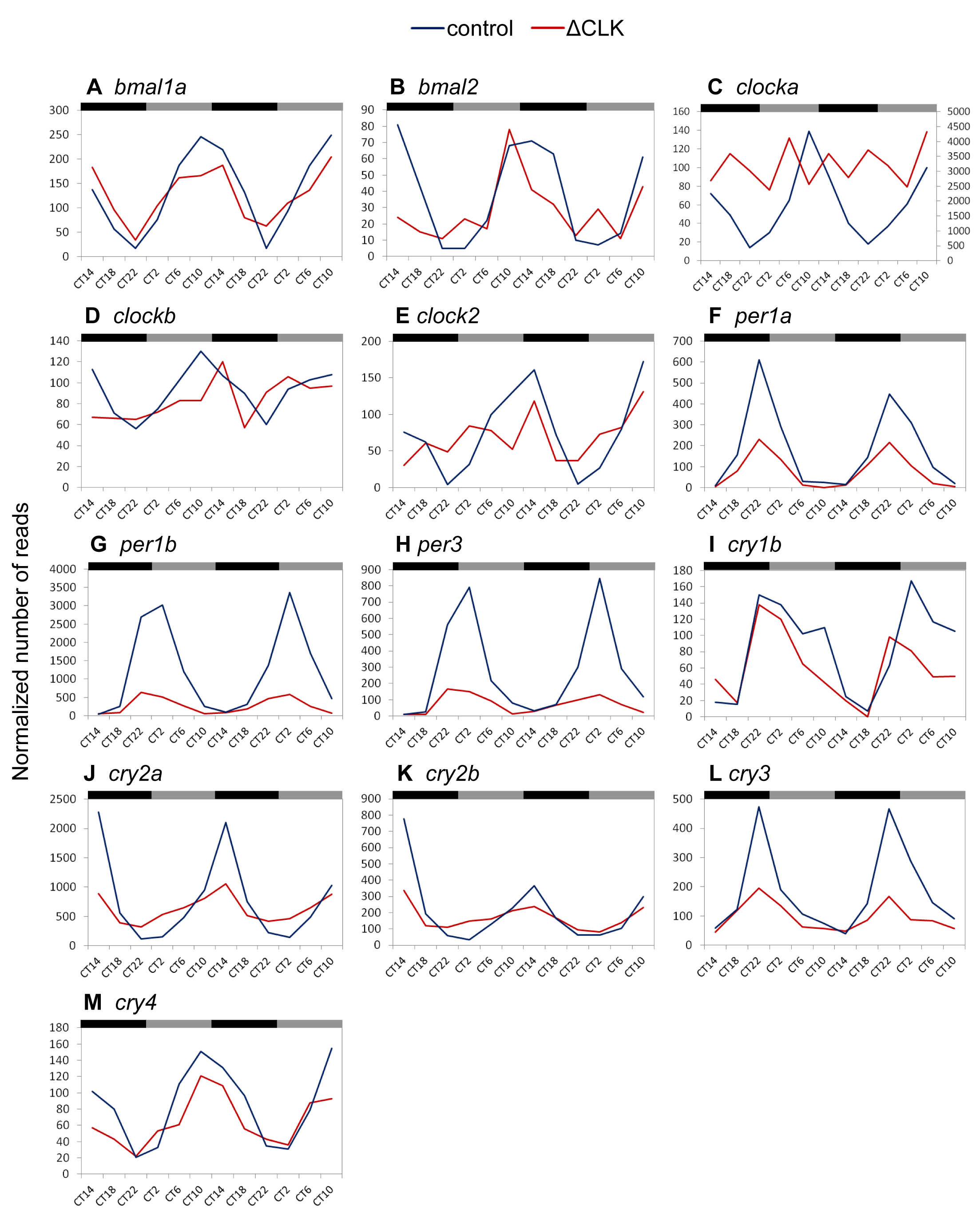

Supplement: S2 Fig — (A–M) Expression profiles of core clock genes that were identified as circadian in the pineal gland of control Tg(aanat2:EGFP) fish (control; blue trendline) compared with their expression profiles in the pineal gland of Tg(aanat2:EGFP-ΔCLK) fish (ΔCLK; red trendline). Black and gray bars denote subjective night and day, respectively. The highly elevated expression of clocka in the Tg(aanat2:EGFP-ΔCLK) pineal gland (C, right vertical axis) reflects overexpression of the transgenic truncated form (ΔCLK). CT, circadian time. The core clock genes per1a, per1b, cry2a and cry3 (F, G, J, L) were identified as circadian in the pineal gland of Tg(aanat2:EGFP-ΔCLK) fish while all other core clock genes lost their circadian rhythmicity, according to Fourier analysis with 90% true-positive rate. (TIF) [file pgen.1006445.s002.tif]

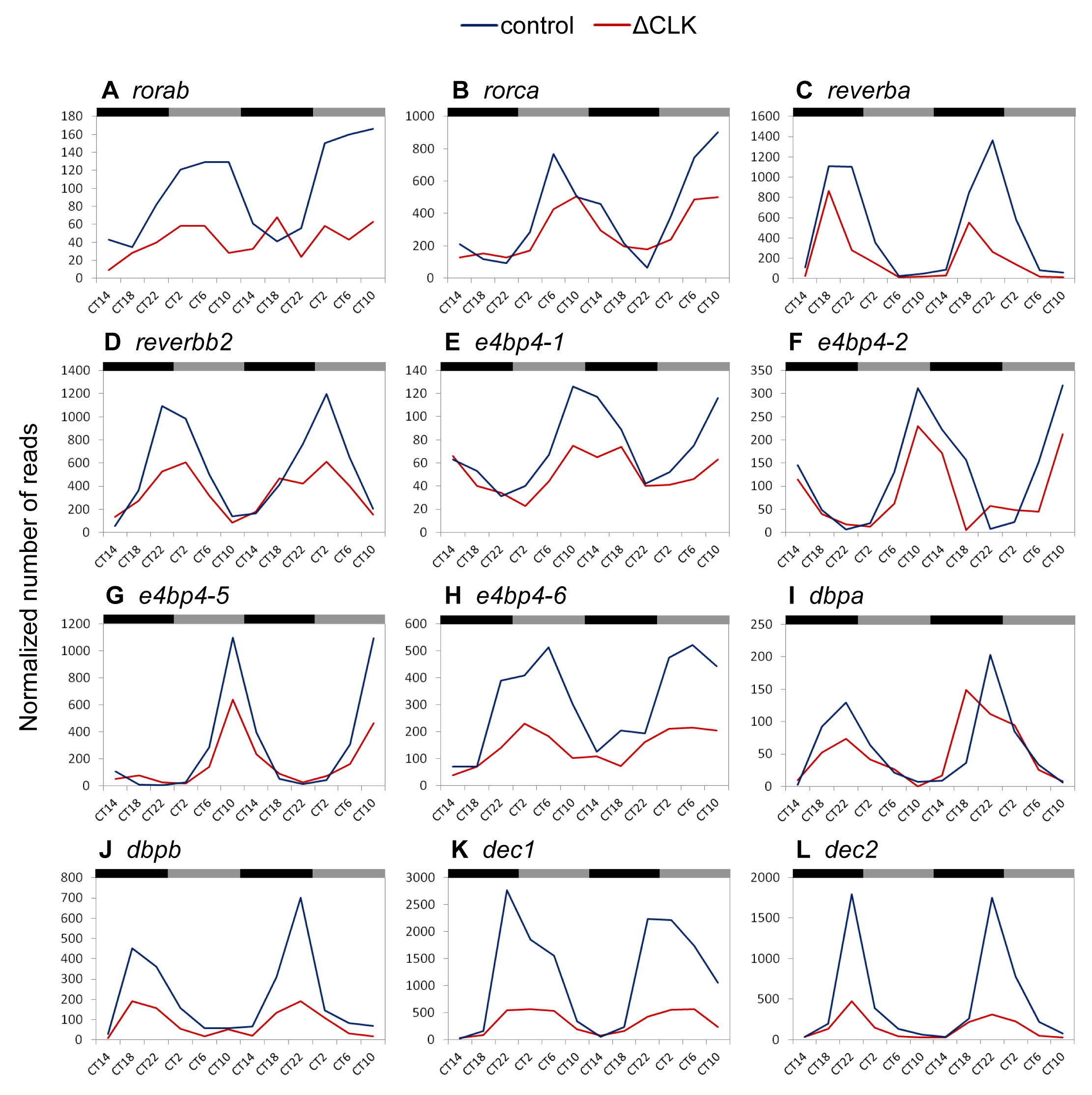

Supplement: S3 Fig — (A–L) Expression profiles of clock-controlled genes that are considered to form accessory loops of the molecular circadian oscillator, and which were identified as circadian in the pineal gland of control Tg(aanat2:EGFP) fish (control; blue trendline), compared with their expression profiles in the pineal gland of Tg(aanat2:EGFP-ΔCLK) fish (ΔCLK; red trendline). Black and gray bars denote subjective night and day, respectively. CT, circadian time. The clock accessory loop genes reverbb2, dec1 and dec2 (D, K, L) were identified as circadian in the pineal gland of Tg(aanat2:EGFP-ΔCLK) fish, whereas, according to Fourier analysis with a 90% true-positive rate, other clock accessory loop genes lost their circadian rhythmicity. (TIF) [file pgen.1006445.s003.tif]

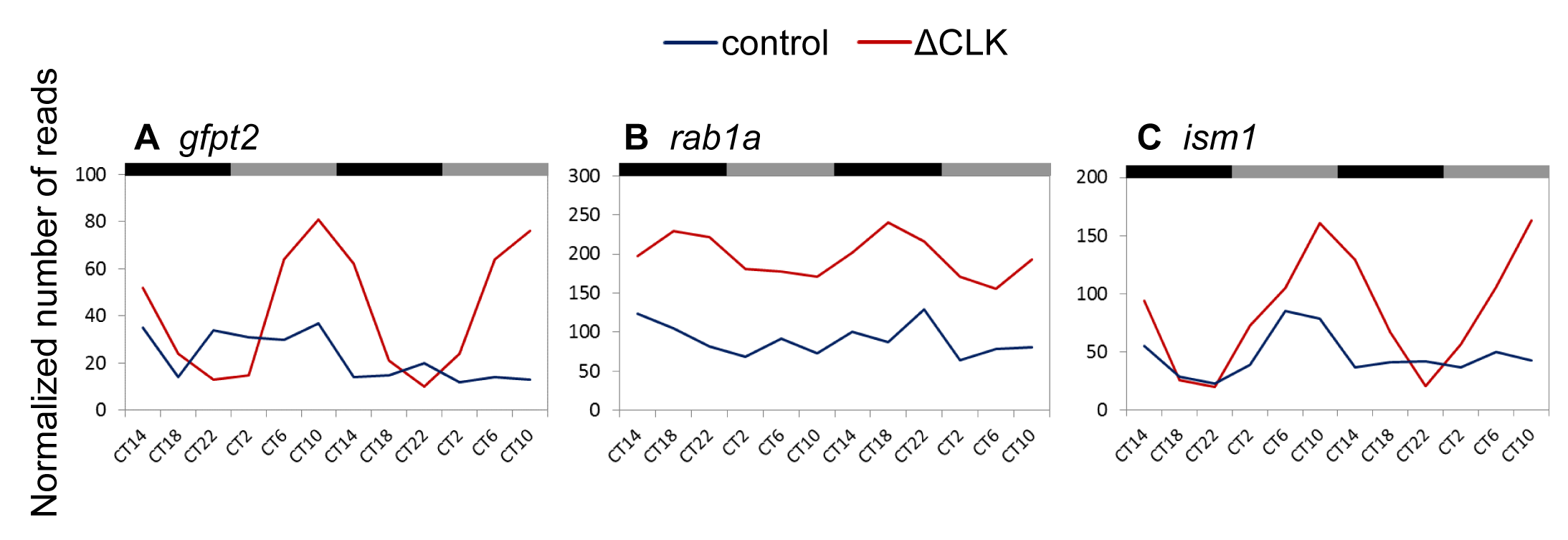

Supplement: S4 Fig — (A–C) Three representative examples of genes that were identified as circadian in the pineal gland of Tg(aanat2:EGFP-ΔCLK) fish (ΔCLK; red trendline) but not in the pineal gland of control Tg(aanat2:EGFP) fish (control; blue trendline). Black and gray bars denote subjective night and day, respectively. CT, circadian time. (TIF) [file pgen.1006445.s004.tif]

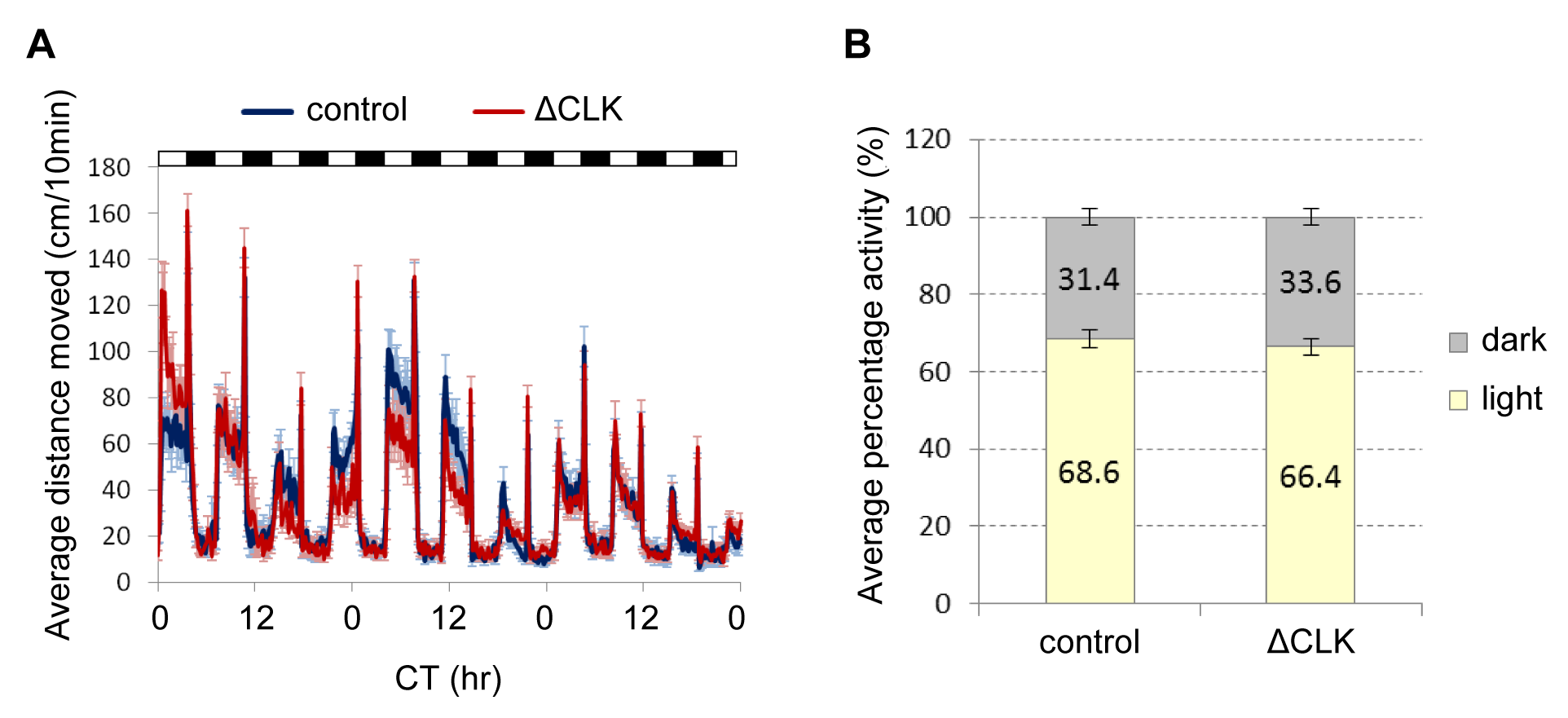

Supplement: S5 Fig — Analysis of locomotor activity of 6–8 dpf Tg(aanat2:EGFP-ΔCLK) larvae (ΔCLK) and control larvae under ten 3.5-hr light: 3.5-hr dark cycles after entrainment by five 12-hr:12-hr LD cycles. (A) The average distance moved (cm/10 min) is plotted on the y-axis and circadian time (CT) on the x-axis. Error bars stand for SE (n = 24). White and black bars represent light and dark, respectively. (B) Average percentage (± SE, n = 24) of activity throughout the bouts of light and dark for each group. No significant difference in the average percentage of activity was found between Tg(aanat2:EGFP-ΔCLK) and control larvae under light and dark conditions. (TIF) [file pgen.1006445.s005.tif]

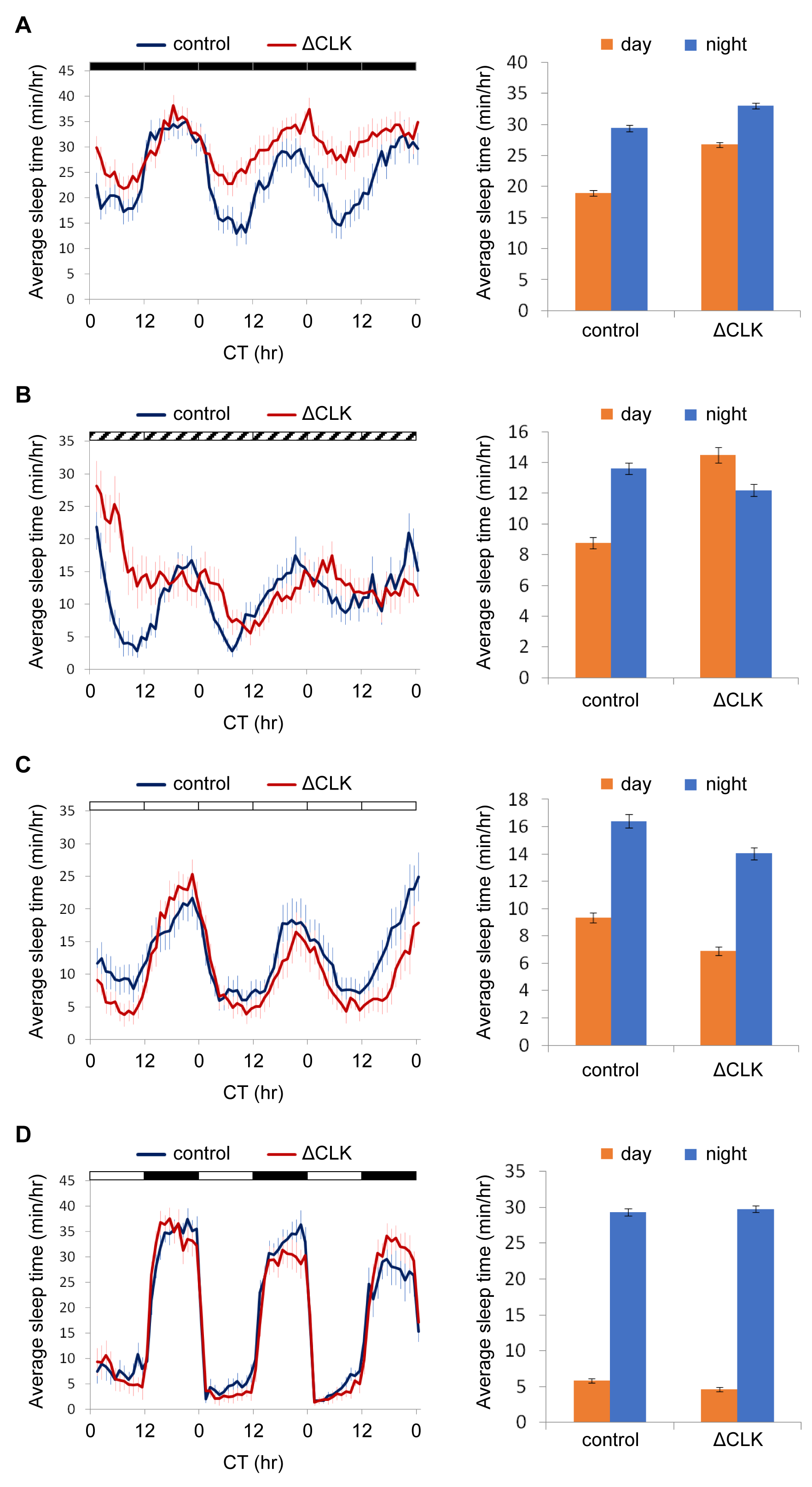

Supplement: S6 Fig — Sleep analysis of 6–8 dpf Tg(aanat2:EGFP-ΔCLK) larvae (ΔCLK) and control larvae under various lighting conditions. A–D, left chart: The average sleep time (min/hr) is plotted on the y-axis and circadian time (CT) on the x-axis. Error bars stand for SE (n = 24); black, white and diagonally lined bars represent dark, light and dim light, respectively. A–D, right chart: The average sleep time (± SE, n = 24) for total subjective daytime and total subjective nighttime for each group. (A) Circadian rhythms of sleep under DD, after entrainment by 5 LD cycles, are affected by blocking the pineal clock; significant differences in day/night sleep time alterations were found between Tg(aanat2:EGFP-ΔCLK) and control larvae (p<0.05, repeated-measures ANOVA). (B) Circadian rhythms of sleep under DimDim, after entrainment by 3 LD and 2 Ldim cycles, are affected by blocking the pineal clock; significant differences in day/night sleep time alterations were found between Tg(aanat2:EGFP-ΔCLK) and control larvae (p<0.0001, repeated-measures ANOVA). (C) Circadian rhythms of sleep under LL, after entrainment by 5 LD cycles, are NOT affected by blocking the pineal clock. (D) Circadian rhythms of sleep under LD cycles are NOT affected by blocking the pineal clock. (TIF) [file pgen.1006445.s006.tif]

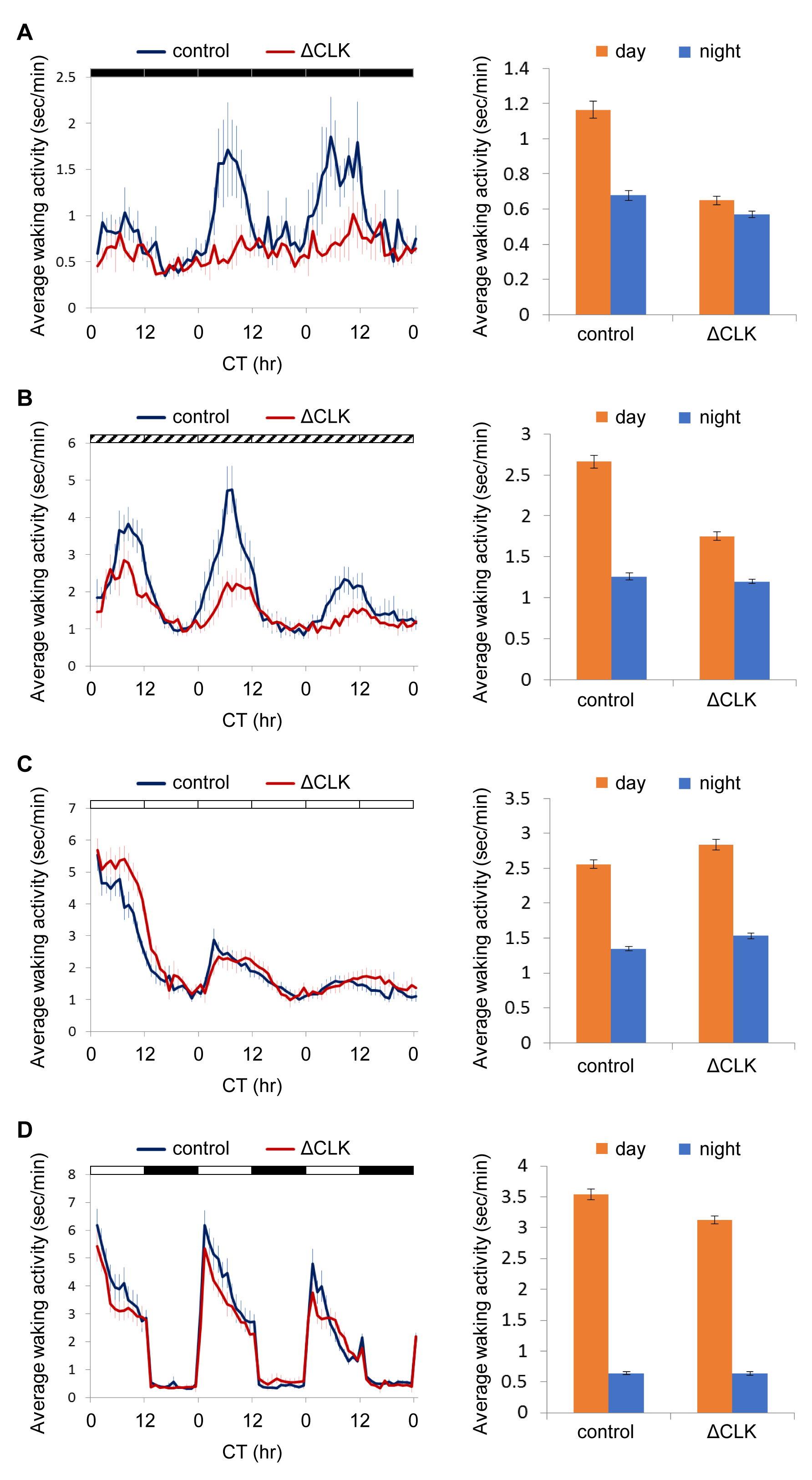

Supplement: S7 Fig — Waking activity analysis of 6–8 dpf Tg(aanat2:EGFP-ΔCLK) larvae (ΔCLK) and control larvae under various lighting conditions. A–D, left chart: The average waking activity (sec/min) is plotted on the y-axis and circadian time (CT) on the x-axis; error bars stand for SE (n = 24); black, white and diagonally lined bars represent dark, light and dim light, respectively. A–D, right chart: The average waking activity (±SE, n = 24) for total subjective daytime and total subjective nighttime for each group. (A) Circadian rhythms of waking activity under DD, after entrainment by 5 LD cycles, are affected by blocking the pineal clock; significant difference in the day/night waking activity alterations was found between Tg(aanat2:EGFP-ΔCLK) and control larvae (p<0.001, repeated-measures ANOVA). (B) Circadian rhythms of waking activity under DimDim, after entrainment by 3 LD and 2 Ldim cycles, are affected by blocking the pineal clock; significant differences in the day/night waking activity alterations were found between Tg(aanat2:EGFP-ΔCLK) and control larvae (p<0.001, repeated-measures ANOVA). (C) Circadian rhythms of waking activity under LL, after entrainment by 5 LD cycles, are NOT affected by blocking the pineal clock. (D) Circadian rhythms of waking activity under LD cycles are NOT affected by blocking the pineal clock. (TIF) [file pgen.1006445.s007.tif]

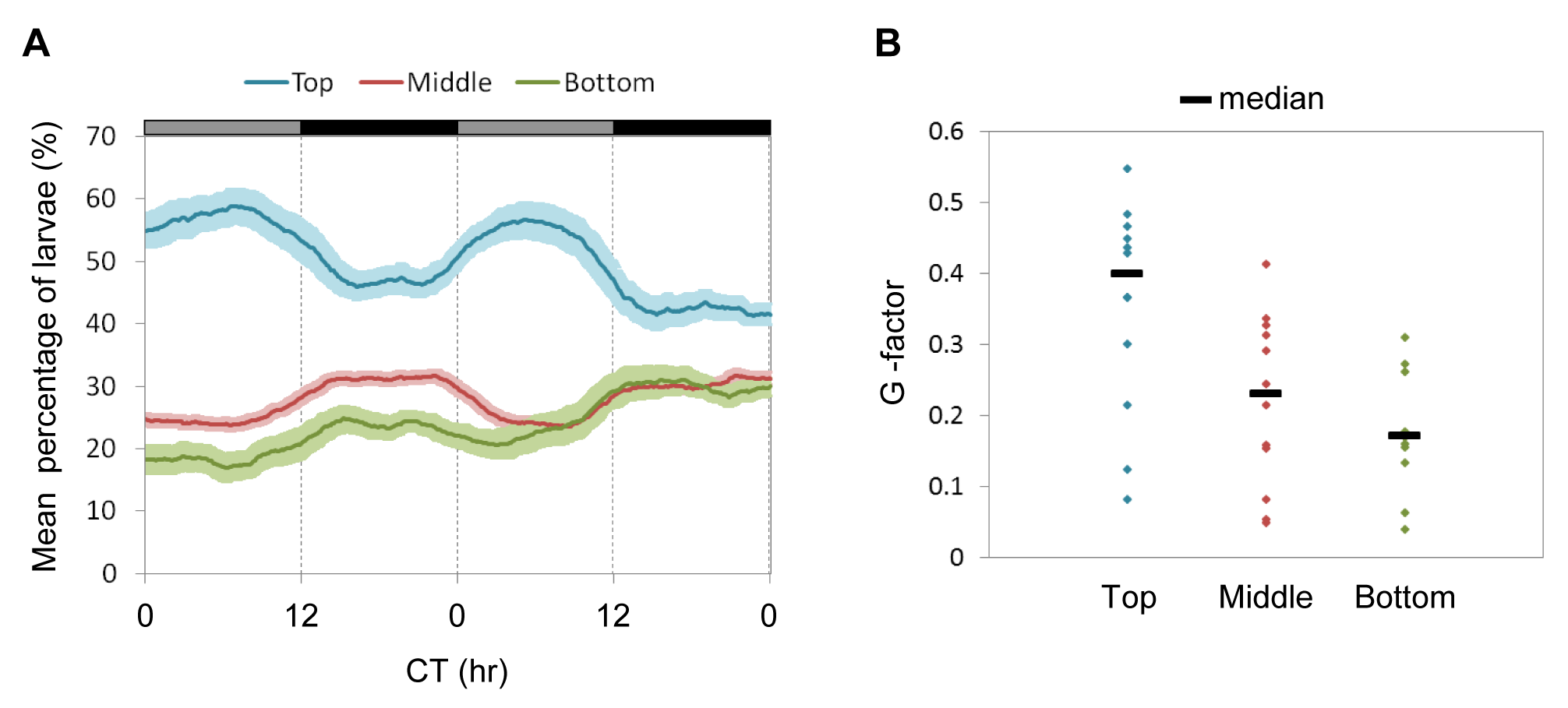

Supplement: S8 Fig — After entrainment to 4 LD cycles, larvae maintained in DD show a daily oscillation in their vertical position in the water column. (A) Mean percentage of larvae in the top, middle and bottom thirds of the water column is plotted on the y-axis and circadian time (CT) on the x-axis. Error bars stand for SE (n = 12). Gray and black horizontal bars represent subjective day and subjective night, respectively. (B) Distribution of the G-factors (see 'Fourier analysis' in S1 Text) of the percentage of larvae in the top, middle and bottom thirds of the water column. The median G-factor values are indicated (black lines). Since the oscillation is most prominent in the top third of the water column, it was selected as the measure for this assay. (TIF) [file pgen.1006445.s008.tif]

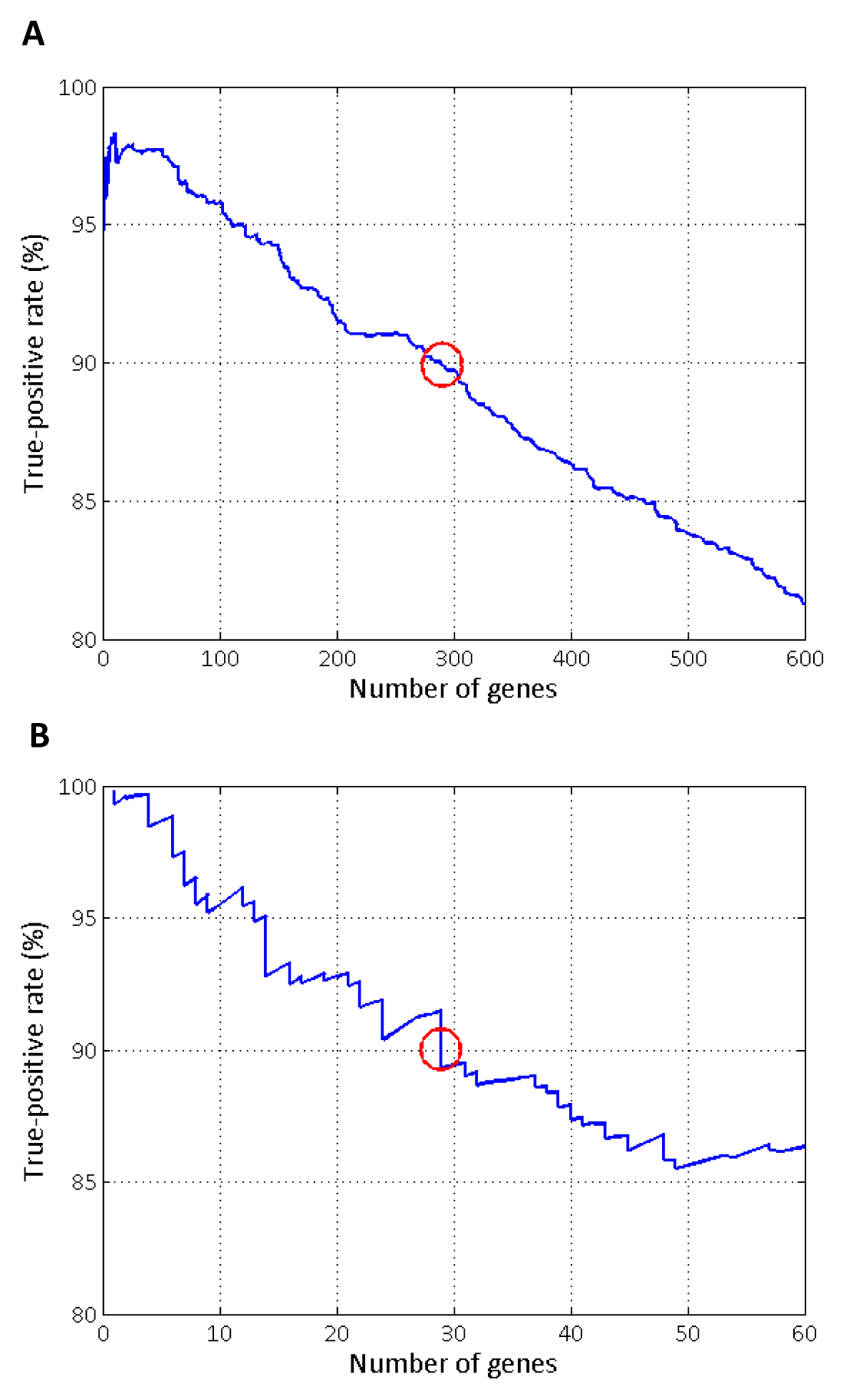

Supplement: S10 Fig — (A) Control Tg(aanat2:EGFP) dataset. (B) Tg(aanat2:EGFP-ΔCLK) dataset. Red circles denote the list length for 90% true-positive rate. (TIF) [file pgen.1006445.s010.tif]
