# Supplementary material for: Genetically Blocking the Zebrafish Pineal Clock Affects Circadian Behavior
Source: PLoS Genet. 2016 Nov 21;12(11):e1006445. doi: 10.1371/journal.pgen.1006445 (PMC5147766; doi:10.1371/journal.pgen.1006445)
Supplement: S9 Fig — Color coding as indicated. (PDF) [file pgen.1006445.s009.pdf]

S9 Fig. Detailed sequence of the *pT2-aanat2:EGFP-2A-Δclocka-5×MYC* transgenic construct. Color coding as indicated.

Underlined: pT2KXIGΔin vector backbone
zebrafish *aanat2* Pineal-Restrictive Downstream Module (PRDM)
1.65 kb of the 5'-flanking region and the 5'-UTR of zebrafish *aanat2*
EGFP CDS
2A peptide linker
ΔCLK CDS (encoding the first 396 amino acids of zebrafish CLOCKa)
5× Myc tag
SV40 poly(A) signal

…CAGAGGTGTAAAAAGTACTCAAAAATTTTACTCAAGTGAAAGTACAAGTACTTAGGGAAAATTTTACTCAATTAAAAGTAAAAGTATC
TGGCTAGAATCTTACTTGAGTAAAAGTAAAAAAGTACTCCATTAAAATTGTACTTGAGTATTAAGGAAGTAAAAGTAAAAGCAAGAAAG
AAAACTAGAGATTCTTGTTTAAGCTTTTAATCTCAAAAAACATTAAATGAAATGCATACAAGGTTTTATCCTGCTTTAGAACTGTTTGT
ATTTAATTATCAAACTATAAGACAGACAATCTAATGCCAGTACACGCTACTCAAAGTTGTAAAACCTCAGATTTAACTTCAGTAGAAGC
TGATTCTCAAAATTGTTAGTGTCAAGCCTAGCTCTTTTGGGGCTGAAAAGCAATCCTGCAGTGCTGAAAAGCCTCTCACAGGCAGCCGA
TGCGGGAAGAGGTGTATTAGTCTTGATAGAGAGGCTGCAAATAGCAGGAAACGTGAGCAGAGACTCCCTGGTGTCTGAAACACAGGCCA
GATGGGCCCCTCTGCTAACCATGTTCATGCCTTCTTCTCTTTCCTACAGCTCCTGGGCAACGTGCTGGTTGTTGTGCTGTCTCATCATT
TTGGCAAAGAATTCCTCGACGGATCCTCGAGACACCGCAGTATATATCACACTCTTCAAAAGCTGGCACATTTGATCAGATTATCTGCC
TTTAGAAACAGCTGACACTCGAAGGAGATGCTGCCGTTCAGCCGGCATGATCTGTAGTAGTAATCAAAGAGAGTGCTACGCTGAGCTGC
ACTTTGGCTCTTTGCTTTTCTTAAGAAGGCTCAGTAGATTAATACCTCGGCACGTGCCTCTGTCACACACCTTCACTATCACGCTTGCA
AACACCCTTGAAGTTGAATTCTGCAGTCGACTCGATCATATTTTCTAGACCTGGTAAGAACTCTGGCAGCTGCATTTTGTACTAATTGA
AGTTCGTTAATTGAGGATGCTGGGCTGCCTGCAAACAGATCATTAGAGTAGTCCAGCCTAGAAGTCATAAAAGTATTGACTAACTTGGA
GCACCATCTGCTGTTAAAATCTATCCTAGAGTGCCACGTGCTGTTAAAAACTGGCATAGCCTAGAGCGCCATCTGCTGTTAAAAGCTAG
CCTAGAATGCCATTTTATGTTAAAAACTAGCCTAATGCACCATCTGCTAGTAAAAACTAAAGTAGCCTAGAACACCATCTGGTGGTAAA
AACTAACCTAGACTGCCACCTTGTGTTAAAAACTAAGGTAGTCCAGAACGCCATCTGGTGGTAAAACCTAGTCTAGAATCTCATTTTCT
GTTAAAACTAGTCTAGAGCACCATCTGCCAGTAAAAACTAACCTGGATTGCCATCTGCTGTTAAAAACTATGGTGGCCTAGAGCACCAA
GTTCTGGTAAAAACTAGAGTAGCCTAGAGCGCCATCTGCTGTTAAAAACTAACCTGGATTGCCATCTGCTGTTAAAAACCAAGTGTAGC
CTAGAGCAACAAGTTCTGGTAAAAACTAGAGTAGCCTAGAGCGCCATGCCCCTGCTAAAAACTAACTTGGATTGCCATGTGCTGTTAAA
ACCCGAGTGTAGCCTAGAGCAACAAGTTCTGGTAAAAACTAGAGTAGCCTAGAGCGCCATCTGCTGTTAAAAACTAACCTGGATTGCCA
TCTGCTGTTAAAAACCAGTAGCCTAGAGCAACAAGTTCTGGTAAAAACTAGAGTAGCCTAGAGCGCCATCTGCTGTTAAAAACTAACCT
GGATTGCCATCTGCTGTTAAAAACCAAGTGTAGCCTAGAGCAACAAGTTCTGGTAAAAACTAGAGTAGCCTAGAGCGCCATCTGCTGTT
AAAAACTAACCTGGATTGCCATCTGCTGTTAAAAACCAAGTGTAGCCTAGAGCAACAAGTTCTGGTAAAAACTAGAGTAGCCTAGAGCG
CCTTCTGCTGTTAAAAACTAACCTGGATTGCCATCTGCTGTTAAAAACCAAGTGTAGCCTAGAGCAACAAGTTCTGGTAAAAACTAGAG
TAGCCTAGAGCACCATCTGCTGTTAAAAACTAAGCTCAGAATCAATTCAAGAGAGCAGTTTGATATTTTTCAGAAGTGATGCAGAAGCA
ATTTCTCAAATGTGTATTTGTCCACAGCTCTAATAGATGTTTTATTTTTTGGAGCTTCATTCCGAAGTTAGGACTATAACACTTCTGTT
AATTGTACCTGTGGGTTTTATTTATCATCTGACAATCTCATCCATGACCCATGACTAGTTCTCACTGTCTTAATTCTCTGCGGCTTGAT
GAATTTGGAAACTGCAGATATGAAAAAGCAAGCAGGACTGGCTTAACCAGCGCAAAGCTTTCTCTGGCTGTACAAACAGTTTCTGGTAA
GTGTTTGGAGAGGCTGATCAGCTGCGCGTTGAGTACCACAGGCTTTCAGCTCACAGACTTTTAGGGAATTGCTGCATCTTCACCTGGCA
CGTGTGCGCACTTTAGGAGATCAGGGGCGGGCTGGAAAACAGACGGGATGAGGGGAGGGTTAAAGGCGAGGAGAGGTGATAGGATTCAA
ACACATCACACCACTGAGACGAGCAGACAAATCCACCAACAGACAGACTGAAGTTTCCTAAAAGTCAGGAGCTCGGGTTAGTTTGGTCA
GTACAGTATTGTCTAAAGTGTGCGCGTGTCAGGATCCACCGGTCGCCACCATGGTGAGCAAGGGCGAGGAGCTGTTCACCGGGGTGGTG
CCCATCCTGGTCGAGCTGGACGGCGACGTAAACGGCCACAAGTTCAGCGTGTCCGGCGAGGGCGAGGGCGATGCCACCTACGGCAAGCT
GACCCTGAAGTTCATCTGCACCACCGGCAAGCTGCCCGTGCCCTGGCCCACCCTCGTGACCACCCTGACCTACGGCGTGCAGTGCTTCA
GCCGCTACCCCGACCACATGAAGCAGCACGACTTCTTCAAGTCCGCCATGCCCGAAGGCTACGTCCAGGAGCGCACCATCTTCTTCAAG
GACGACGGCAACTACAAGACCCGCGCCGAGGTGAAGTTCGAGGGCGACACCCTGGTGAACCGCATCGAGCTGAAGGGCATCGACTTCAA
GGAGGACGGCAACATCCTGGGGCACAAGCTGGAGTACAACTACAACAGCCACAACGTCTATATCATGGCCGACAAGCAGAAGAACGGCA
TCAAGGTGAACTTCAAGATCCGCCACAACATCGAGGACGGCAGCGTGCAGCTCGCCGACCACTACCAGCAGAACACCCCCATCGGCGAC
GGCCCCGTGCTGCTGCCCGACAACCACTACCTGAGCACCCAGTCCGCCCTGAGCAAAGACCCCAACGAGAAGCGCGATCACATGGTCCT
GCTGGAGTTCGTGACCGCCGCCGGGATCACTCTCGGCATGGACGAGCTGTACAAGGGCTCCGGAGCCACGAACTTCTCTCTGTTAAAGC
AAGCAGGAGACGTGGAAGAAAACCCCGGTCCTATGACCTCCAGCATAGACCGGGATGACAGCAGTATCTTTGATGGGTTAATGGAAGAA
GATGAGAAGGACAAAGCAAAAAGAGTTTCTAGAAATAGGTCGGAGAAGAAGAGGCGAGACCAGTTCAATGTGCTCATCAAGGAACTGGG
CACCATGTTGCCGGGCAACACCCGCAAGATGGACAAGTCTACCATCCTACAGAAGAGCATCGACTTCCTGCGCAAGCACAAAGAAATTG
CTGCACAGTCGGAGTCGAGTGAAATCCGTCAGGACTGGAAGCCACCCTTCCTTAGCAATGAGGAGTTCACACAGCTGATGCTGGAGGCA

CTAGATGGCTTCTTTCTGGCCATTATGACGGATGGAAACATAATCTACGTCTCAGAAAGTGTCACGTCTTTATTAGAACATCTACCTTC
TGATCTTGTGGATCAGAACCTGTTGAATTTCCTTCCACTGGGTGAGCATTCGGAGGTGTATAAAGCTCTGTCAACACACATGTTGGAGG
GAGAAACCCTCACCCCAGACTACCTAAAGACAAAGAACCAGTTGGAGTTTTGTTGCCACATGCTCCGGGGCACCATCGACCCCAAAGAG
CCGCCCGTTTATGAATATGTCAAGTTCATCGGAAACTTTAAGTCCCTCAACACTGTGCCTAACTCAACACGAAACGGCTTTGAGGGAGT
GATCCAGCGATCGCTGCGACACGCGTTTGAAGACCGAGTGTGTTTCATAGCGACGGTCAGGCTAGCCAAACCTCAGTTTATCAAGGAAA
TGTGTACTGTGGAGGAGCCCAATGAGGAATTCACCTCCAGACACAGTTTAGAGTGGAAATTCCTCTTCTTGGACCACAGGGCGCCTCCT
ATCATTGGTTACCTGCCGTTTGAGGTTCTGGGTACTTCAGGGTACGACTACTATCATGTGGATGACCTGGAGACTTTGGCCAAATGCCA
CGAACACTTGATGCAGTACGGTAAGGGGAAGTCGTGCTATTACCGTTTTCTGACTAAAGGTCAGCAGTGGATCTGGCTTCAGACGCACT
ACTACATCACCTACCACCAGTGGAACTCAAGGCCAGAGTTCATCGTCTGCACTCACACGGTGGTCAGTTATGCTGAAGTGAGAGCTGAA
CAGCGCAGAGAGCTGGGCATTGAAGAATCACCTCCTGAGATCTCCGCAGACAAGTCTCAGGACTGCGATCGATTTAAAGCTATGGAGCA
AAAGCTCATTTCTGAAGAGGACTTGAATGAAATGGAGCAAAAGCTCATTTCTGAAGAGGACTTGAATGAAATGGAGCAAAAGCTCATTT
CTGAAGAGGACTTGAATGAAATGGAGCAAAAGCTCATTTCTGAAGAGGACTTGAATGAAATGGAGCAAAAGCTCATTTCTGAAGAGGAC
TTGAATGAAATGGAGAGCTTGGGCGACCTCACCATGTGACATATGATGATCCAGACATGATAAGATACATTGATGAGTTTGGACAAACC
ACAACTAGAATGCAGTGAAAAAAATGCTTTATTTGTGAAATTTGTGATGCTATTGCTTTATTTGTAACCATTATAAGCTGCAATAAACA
AGTTAACAACAACAATTGCATTCATTTTATGTTTCAGGTTCAGGGGGAGGTGTGGGAGGTTTTTTAAAGCAAGTAAAACCTCTACAAAT
GTGGTATGGCTGATTATGATCCTCTAGATCAGATCTGCGAAGATACGGCCACGGGTGCTCTTGATCCTGTGGCTGATTTTGGACTGTGC
TGCTCGCAGCTGCTGATGAATCACATACTTCCTCCATTTTCTTCCACTGATTGACTGTTATAATTTCCCTAATTTCCAGGTCAAGGTGC
TGTGCATTGTGGTAATAGATGTGACATGACGTCACTTCCAAAGGACCAATGAACATGTCTGACCAATTTCATATAATGTGAAAACGATT
TTCATAGGCAGAATAAATAACATTTAAATTAAACTGGGCATCAGCGCAATTCAATTGGTTTGGTAATAGCAAGGGAAAATAGAATGAAG
TGATCTCCAAAAAATAAGTACTTTTTGACTGTAAATAAAATTGTAAGGAGTAAAAAGTACTTTTTTTTCTAAAAAAATGTAATTAAGTA
AAAGTAAAAGTATTGATTTTTAATTGTACTCAAGTAAAGTAAAAATCCCCAAAAATAATACTTAAGTACAGTAATCAAGTAAAATTACT
CAAGTACTTTACACCTCTG...
