# Supplementary material for: Genetically Blocking the Zebrafish Pineal Clock Affects Circadian Behavior
Source: PLoS Genet. 2016 Nov 21;12(11):e1006445. doi: 10.1371/journal.pgen.1006445 (PMC5147766; doi:10.1371/journal.pgen.1006445)
Supplement: S1 Text — (PDF) [file pgen.1006445.s013.pdf]

Supplementary Methods

Fourier analysis

The time-dependent signal was converted into a frequency-dependent signal using Fast Fourier Transform (FFT). The analysis was performed separately on the datasets obtained from the Tg(*aanat2*:EGFP-ΔCLK) fish and the control Tg(*aanat2*:EGFP) fish (data from Tovin et al. [1]). The extent to which the original signal is circadian was quantified by the ratio ('G-factor') of the power (squared amplitude) of the frequency corresponding to a 24-hr period to the sum of powers of all frequencies [1,2]. The higher the G-factor, the higher the confidence that the transcript is circadian. We observed that changing the definition of the G-factor by adding the powers of higher harmonics of the 24-hr period to the numerator gave similar results to those obtained according to the above definition. To determine the true-positive rate for a list of transcripts constructed using a given G-factor cutoff, we conducted a permutation analysis as follows: (1) The time-dependent signals of each transcript were randomly shuffled. (2) FFT was performed and the G-factor was calculated for each transcript. (3) The cumulative histogram of the G-factor values (ranging from 0 to 1) was calculated, yielding the number of transcripts whose calculated G-factor is larger than a given value ('random detection function'). (4) Steps 1−3 were repeated a thousand times and the averaged random detection function was calculated. This function estimates the number of false-positive detections of circadian genes for any given cutoff value of the G-factor. (5) Steps 2−3 were performed on the original dataset. The resulting function, providing the number of transcripts exhibiting a G-factor larger than a given value in the real data, was termed the 'detection function'. (6) For any given choice of cutoff for the G-factor, the difference between the detection function and the average random detection function estimates the number of true-positives in the list of transcripts constructed with this cutoff. Subsequently, using the number of transcripts detected for a given G-factor (step 5) and the number of true-positives for a given G-factor (step 6), the true-positive rate was calculated as a function of the number of transcripts

detected, and was further used to identify circadian genes with high accuracy (S10 Fig). A true-positive rate of at least 90% corresponds to a G-factor of ~0.80 and above in the Tg(*aanat2*:EGFP-ΔCLK) fish dataset and to a G-factor of ~0.71 and above in the control Tg(*aanat2*:EGFP) fish dataset. The procedure described here was implemented using in-house MATLAB (The MathWorks, Inc.) script.

Comparison of pineal gland samples from CT2 and CT14b between the different experiments

The data of control pineal gland samples from Tg(*aanat2*:EGFP) fish, taken at CT2 and CT14b, were utilized to compare the present sequencing experiment with the one performed by Tovin et al. [1]. To that end we detected genes with large differences in expression levels (≥2-fold) between CT2 and CT14b, requiring that in at least one of these time points the expression level was in the upper quartile of all transcripts. This list of genes was compared with two additional lists, generated from the same time points and with the same cutoffs, using data from Tg(*aanat2*:EGFP) fish that were previously reported by Tovin et al. [1] and from Tg(*aanat2*:EGFP-ΔCLK) fish. As expected, the overlap between the lists was highest when the current data of Tg(*aanat2*:EGFP) fish were compared with the data obtained from the previous experiment with Tg(*aanat2*:EGFP) fish by Tovin et al. [1], and less high when the current data of Tg(*aanat2*:EGFP) fish were compared with the data obtained from Tg(*aanat2*:EGFP-ΔCLK) fish, or when the data from Tg(*aanat2*:EGFP-ΔCLK) fish were compared with the data from the previous experiment with Tg(*aanat2*:EGFP) fish, with 161, 62 and 76 overlapping genes, respectively, thereby validating the comparison between the current and the previously [1] collected data.

Transient transfection of Pac-2 cells and real-time bioluminescence assay

Pac-2 cells [3] were cultured as described [4] and transiently co-transfected with ΔCLK and *E-box*$_{per1b}$*-Luc* using the FuGene HD reagent (Roche Diagnostics) according to the manufacturer's

protocol. The enhancer-reporter construct *E-box*$_{per1b}$*-Luc* [5] contains four copies of the *per1b* E-box (5'-GAAGCACGTGTACTCG-3') cloned into the minimal promoter luciferase reporter vector pLucMCS (Stratagene). The coding sequence of ΔCLK was incorporated into the CMV promoter-driven expression vector pCS2-MTK (derivative of pCS2; [6]). An empty expression vector served as negative control. Transiently transfected cells were exposed to three LD cycles and two daily cycles under DD, and a real-time bioluminescence assay was carried out as described elsewhere [5,7] using a TopCount NXT scintillation counter (2-detector model; Packard). Traces represent the mean of four independently transfected wells ± SD. Short-term and long-term trends were removed from the raw data by an adjacent-averaging method with 8-hr and 5-day running means, respectively. Normalized values were obtained by dividing the bioluminescence values by their average value.

Supplementary References

1. Tovin A, Alon S, Ben-Moshe Z, Mracek P, Vatine G, et al. (2012) Systematic identification of rhythmic genes reveals camk1gb as a new element in the circadian clockwork. PLoS Genet 8: e1003116.
2. Levy O, Kaniewska P, Alon S, Eisenberg E, Karako-Lampert S, et al. (2011) Complex diel cycles of gene expression in coral-algal symbiosis. Science 331: 175.
3. Lin S, Gaiano N, Culp P, Burns JC, Friedmann T, et al. (1994) Integration and germ-line transmission of a pseudotyped retroviral vector in zebrafish. Science 265: 666-669.
4. Vallone D, Santoriello C, Gondi SB, Foulkes NS (2007) Basic protocols for zebrafish cell lines: maintenance and transfection. Methods Mol Biol 362: 429-441.
5. Vallone D, Gondi SB, Whitmore D, Foulkes NS (2004) E-box function in a period gene repressed by light. Proc Natl Acad Sci U S A 101: 4106-4111.
6. Travnickova-Bendova Z, Cermakian N, Reppert SM, Sassone-Corsi P (2002) Bimodal regulation of mPeriod promoters by CREB-dependent signaling and CLOCK/BMAL1 activity. Proc Natl Acad Sci U S A 99: 7728-7733.
7. Vatine G, Vallone D, Appelbaum L, Mracek P, Ben-Moshe Z, et al. (2009) Light directs zebrafish period2 expression via conserved D and E boxes. PLoS Biol 7: e1000223.
